# Sub-Cellular Localization and Complex Formation by Aminoacyl-tRNA Synthetases in Cyanobacteria: Evidence for Interaction of Membrane-Anchored ValRS with ATP Synthase

**DOI:** 10.3389/fmicb.2016.00857

**Published:** 2016-06-06

**Authors:** Javier Santamaría-Gómez, Jesús A. G. Ochoa de Alda, Elvira Olmedo-Verd, Roque Bru-Martínez, Ignacio Luque

**Affiliations:** ^1^Instituto de Bioquímica Vegetal y Fotosíntesis, Consejo Superior de Investigaciones Científicas and Universidad de SevillaSeville, Spain; ^2^Facultad de Formación del Profesorado, Universidad de ExtremaduraCáceres, Spain; ^3^Department of Agrochemistry and Biochemistry, Faculty of Science, University of AlicanteAlicante, Spain

**Keywords:** aminoacyl-tRNA synthetases, membrane-anchoring, cyanobacteria, FoF1 ATP synthase, thylakoids, CAAD

## Abstract

tRNAs are charged with cognate amino acids by aminoacyl-tRNA synthetases (aaRSs) and subsequently delivered to the ribosome to be used as substrates for gene translation. Whether aminoacyl-tRNAs are channeled to the ribosome by transit within translational complexes that avoid their diffusion in the cytoplasm is a matter of intense investigation in organisms of the three domains of life. In the cyanobacterium *Anabaena* sp. PCC 7120, the valyl-tRNA synthetase (ValRS) is anchored to thylakoid membranes by means of the CAAD domain. We have investigated whether in this organism ValRS could act as a hub for the nucleation of a translational complex by attracting other aaRSs to the membranes. Out of the 20 aaRSs, only ValRS was found to localize in thylakoid membranes whereas the other enzymes occupied the soluble portion of the cytoplasm. To investigate the basis for this asymmetric distribution of aaRSs, a global search for proteins interacting with the 20 aaRSs was conducted. The interaction between ValRS and the FoF1 ATP synthase complex here reported is of utmost interest and suggests a functional link between elements of the gene translation and energy production machineries.

## Introduction

A hallmark of life is the reclusion of molecules in individual entities named cells with an uneven distribution of components within the internal space. Internal organization of cells is complex and it is optimized for functioning. Components are confined to a particular cell structure or compartment either because such environment is best suited for its function, to make possible their interaction with other components (i.e., substrates, regulators) or to avoid spurious interactions with other molecules (Yeates et al., 2008; Shapiro et al., 2009; Montero Llopis et al., 2010). Compared to eukaryotic cells, bacteria are structurally simple as they lack a nucleus and organelles. Cyanobacteria are unique among bacteria in the sense that they posses an intracytoplasmic system of membrane saculi named thylakoids, which act as a physical support for large protein complexes involved in energy production by photosynthesis, including photosystems I and II, cytochrome *b*_6_*f* and ATP synthase (Liberton and Pakrasi, 2008). Recent investigations have demonstrated that in some cyanobacteria components of the gene translation machinery, namely aminoacyl-tRNA synthetases, are also anchored to thylakoid membranes (Olmedo-Verd et al., 2011).

Gene translation in the cytoplasm of bacteria occurs at 70S ribosomes through pairing the anticodons of charged tRNAs to complementary codons of mRNAs. Charged tRNAs or aminoacyl-tRNAs are produced by aminoacyl-tRNA synthetases (aaRSs), a family of enzymes that catalyze the ATP-dependent esterification of amino acids to the 3′ acceptor end of cognate tRNAs (Ibba and Soll, 2000). Cells generally count with a set of twenty aaRSs, each one specific for its cognate amino acid. According to structural and conservation criteria, aaRSs are partitioned in two classes with distinct phylogenetic origins (Eriani et al., 1990; Ribas de Pouplana and Schimmel, 2001). Once produced, aminoacyl-tRNAs form a complex with GTP-activated elongation factor-Tu that delivers them to the A site of the ribosome (Simonetti et al., 2009).

A striking trait of species of the phylum Cyanobacteria observed in no other living organism is the presence of catalytically-active aaRSs anchored to membranes (Luque et al., 2008; Olmedo-Verd et al., 2011). Membrane anchoring was particularly well demonstrated for the Valyl-tRNA synthetase (ValRS) of *Anabaena* sp. PCC 7120 (hereafter *Anabaena*), which harbors an idiosyncratic domain, termed CAAD, with a putative C-terminal coiled-coil and two transmembrane helices, that mediates its binding to the lipid bilayer (Luque et al., 2008; Olmedo-Verd et al., 2011; Luque and Ochoa de Alda, 2014). This domain was also observed in the ValRS of some other cyanobacterial species. Interestingly, in few other species, CAAD was found inserted either in GluRS, IleRS, or LeuRS (Luque et al., 2008). CAAD is homologous to a family of stand-alone membrane proteins of cyanobacteria and plant chloroplast, named Curt1, or CURT1, respectively (Armbruster et al., 2013; Luque and Ochoa de Alda, 2014). Cyanobacteria and plant chloroplasts share a common phylogenetic origin, they both perform oxygenic photosynthesis and show functional and structural similarities. Thylakoid membranes are distributed in the stroma of chloroplasts or the cytoplasm of cyanobacteria, which are topologically equivalent. In chloroplasts, CURT1 proteins are integral membrane proteins that localize at the edges of thylakoid grana saculi and are responsible for the pronounced curvature of the membrane at this position (Armbruster et al., 2013). Cyanobacteria thylakoids do not pile up forming grana, however, *Arabidopsis* CURT1A was able to partially complement a *curt1* mutant of the cyanobacterium *Synechocystis* sp. PCC 6803, indicating some degree of functional conservation (Armbruster et al., 2013).

CAAD mediates the specific targeting of ValRS to thylakoid membranes in *Anabaena* (Olmedo-Verd et al., 2011). Consistent with the absence of any ValRS paralog in *Anabaena*, tRNA^Val^-charging activity is confined to the membrane fractions of cell extracts. Artificial removal of CAAD from *Anabaena* ValRS rendered the enzyme soluble but had little impact on its catalytic parameters, indicating that CAAD's function is mostly structural (Olmedo-Verd et al., 2011). Based on these data, it was proposed that in the other species where ValRS was also observed to contain CAAD, the enzyme would localize in thylakoid membranes. Furthermore, it was assumed that in the species where GluRS, IleRS, or LeuRS contained such domain, these enzymes would share such subcellular localization, but this has not been demonstrated. Conversely, aaRSs lacking CAAD in *Anabaena* and other species were all assumed to be soluble, which for the vast majority of them still awaits empirical demonstration. Worth to investigate is the possibility that despite the absence of an apparent membrane-anchoring domain, some of these aaRSs may be indirectly attached to membranes through interaction with membrane proteins. It has been shown that aaRSs form stable or transient complexes with other proteins in a variety of organism, a phenomenon that appears frequent in eukaryotes and archaea and less common in bacteria, where aaRSs are thought to be dispersed in the cytoplasm (Mirande et al., 1982; Hausmann and Ibba, 2008; Godinic-Mikulcic et al., 2011; Raina et al., 2012; Laporte et al., 2014).

Notwithstanding the recent demonstration of the anchoring of the ValRS to thylakoid membranes in *Anabaena*, the information on the sub-cellular localization of aaRSs in cyanobacterial cells is limited and a global picture of the distribution of the full complement of aaRSs is lacking. This information is fundamental to understand how the initial steps of translation occur in these organisms and whether they are confined to a particular location. The aim of this work was to determine the sub-cellular localization for all aaRSs in *Anabaena* and to investigate the biological basis for such subcellular distribution. As an approach for this latter goal, we undertook the identification of interacting partners for each aaRSs. In this work we present compelling evidence on the interaction of the membrane-anchored ValRS with the FoF1-ATP synthase complex in *Anabaena* and we identify proteins interacting with soluble aaRSs in this organism.

## Materials and methods

### Organisms and growth conditions

*Anabaena* sp. PCC 7120 and derivative strains were grown under standard growth conditions [30°C, continuous illumination (75 μE m^−2^ s^−1^) and bubbled with a mixture of CO_2_ and air (1% v/v) in BG11 medium (Rippka, 1988) supplemented with 10 mM NaHCO_3_]. For the preparation of solid media Difco agar was added at a final concentration of 1% (w/v). When required, antibiotics were used at the following concentrations: neomycin, 10 μg ml^−1^ for liquid media and 50 μg ml^−1^ for solid media; streptomycin 2 μg ml^−1^ for liquid media and 5 μg ml^−1^ for solid media; spectinomycin 2 μg ml^−1^ for liquid media and 5 μg ml^−1^ for solid media. In experiments where heterocysts were induced to differentiate, cells from bubbled cultures in BG11 medium were filtered, washed twice with BG11_0_ medium (similar to BG11 but lacking NaNO_3_), inoculated in BG11_0_ medium supplemented with 10 mM NaHCO_3_, and cultured for further 24 h at 30°C under continuous illumination. When indicated, cultures were supplemented with L-methionine sulfoximine (MSX) at a final concentration 2.5 μM.

*Escherichia coli* was cultured in LB medium supplemented with antibiotics at standard concentrations when necessary (Ausubel et al., 2010). Strain DH5α was used for regular cloning and strain C41(DE3) was used for the expression of *Anabaena* ValRS::His and ValRSΔ^C^::His proteins under the control of the T7 promoter. Expression of the T7 RNA polymerase in C41(DE3) cells was induced by addition of IPTG (isopropyl β-D-1-thiogalactopyranoside) at a final concentration of 0.4 mM.

### Cell fractionation

Cyanobacterial cell fractionation and membrane preparation was performed following a procedure based on that described by Sobotka et al. (2008), with some modifications (Olmedo-Verd et al., 2011). Cells from 600 to 700 ml cultures of cyanobacteria were harvested by centrifugation, washed with 50 mM Tris-HCl buffer [pH 7.5] and resuspended in buffer T (20 mM HEPES-NaOH [pH 7.5], 10 mM MgCl_2_, 5 mM CaCl_2_ and 20% glycerol) at a ratio of 5 ml of buffer T per gram of cell pellet (wet weight). Cells were disrupted in a French press at 9000 psi in the presence of 1 mM PMSF (phenylmethylsulfonyl fluoride) and *Complete*™ EDTA free, (Sigma) protease inhibitor cocktail. The cell extracts were centrifuged at 32,000 x g for 10 min at 4°C to eliminate unbroken cells and cell debris. The supernatant (referred to as the “cell extract”) was ultracentrifuged at 100,000 x g for 1 h at 4°C. To avoid contamination with the membrane fraction only the top portion (about 75% of the total volume) of the supernatant containing the soluble fraction was saved, while the bottom portion in direct contact with the pellet containing the membranes was discarded. The pellet containing the membranes was washed with buffer T and resuspended in the same buffer supplemented with 1% n-dodecyl-β-D-maltoside. In experiments where membrane purity was paramount, pellets were resuspended in buffer T and ultracentrifuged a second time at 100,000 x g for 1 h at 4°C. The supernatant was discarded and the pellet was resuspended in the same buffer supplemented with 1% n-dodecyl-β-D-maltoside. The chlorophyll concentration in cyanobacterial cultures or cell fractions were determined as previously described (Mackinney, 1941), while the protein content was determined by the modified Lowry procedure (Markwell et al., 1978).

Fractionation of IPTG-induced *E. coli* C41(DE3) cells was carried out by resuspension of cells in buffer A (0.05 M phosphate buffer [pH 8], 0.15 M NaCl, and 10% glycerol) and disruption in the French press at 12,000 psi in the presence of 1 mM PMSF. Extracts were subjected to a first centrifugation at 21,000 x g and the supernatant to a second centrifugation at 100,000 x g. The supernatant from the second centrifugation contained the soluble fraction. The pellet corresponding to the membrane fraction was washed extensively with buffer A and resuspended in buffer A supplemented with 1% n-dodecyl-β-D-maltoside.

The plasmid and strain construction is described in Table S5. The oligonucleotides used are listed in Table S6.

### Colorless-native page (CN-PAGE)

*Anabaena* cells were cultured in BG11-Cu^2+^ (BG11 medium omitting CuCl_2_) under standard laboratory conditions till they reached a chlorophyll concentration of 4–5 μg/ml. At this point, CuSO_4_ was added at a final concentration of 1.5 μM to induce the expression of the *petE* promoter and cells were further cultured under the same conditions for 24 h.

Cells from 700 ml cultures were harvested by filtration washed with thylakoid buffer (25 mM MES-NaOH pH 6.5, 5 mM CaCl_2_, 10 mM MgCl_2_, 20% glycerol) and resuspended in the same buffer at a ratio of 5 ml of buffer per gram of cells. Cells were disrupted after the addition of protease inhibitors (*Complete*™ EDTA free, Sigma) by three passages through a French press at 9000 p.s.i. and extracts were fractionated according to Kopecna et al. (2012). The chlorophyll content of the membrane suspension was measured, digitonin was added at a ratio of 10 grams per gram of chlorophyll, and incubated at 4°C for 30 min.

Samples containing 5 μg of chlorophyll were first resolved in a 3–13% polyacrylamide gel containing 0.005% digitonin (Krause and Seelert, 2008). The gel was cut into portions corresponding to each lane incubated for 1 h in denaturing solution containing 2% SDS, 66 mM Na_2_CO_3_, and 0.67% 2-mercaptoethanol. Each gel piece was placed on a denaturing (8–20%) polyacrylamide gel and subjected to SDS-PAGE. Proteins were visualized by gel staining with Coomassie blue.

### *In vivo* cross-linking and protein purification

*Anabaena* was cultured in BG11 or BG11-Cu^2+^medium under standard conditions to a chlorophyll concentration of 4–5 μg/ml. In those cases were induction of the *petE* promoter was needed CuSO_4_ was added to a final concentration of 1.5 μM and cells were further cultured for 24 h. Cross-linking was performed as described (Staron et al., 2011). Briefly, a solution containing 4% formaldehyde in PBS buffer (10 mM Na_2_HPO_4_, 1.8 mM KH_2_PO_4_, 137 mM NaCl, 2.7 mM KCl) was added to cultures so that the final concentration of formaldehyde was 0.5% and were incubated under standard culture conditions for 20 min at room temperature. Cells from 80 ml cultures were harvested by centrifugation at 4500 x g for 5 min at 4°C, washed with 100 mM Tris-HCl buffer pH 8 and resuspended in the same buffer at a ratio of 2 ml of buffer per gram of the cell pellet. Protease inhibitor (*Complete*™ EDTA free, Sigma) was added and cells were disrupted by 10 passages through a French press at 18000 psi. Extracts were fractionated by centrifugation at 32000 x g for 15 min at 4°C. The pellet containing unbroken cells and cell debris was discarded and the supernatant was ultracentrifugated at 100,000 x g for 1 h at 4°C. The supernatant corresponding to the soluble fraction was carefully transferred to new tubes and the pellets corresponding to the membrane fraction was washed three times with 100 mM Tris-HCl buffer pH 8. Pellets were resuspended by addition of 500 μl of lysis buffer (150 mM NaCl, 1% Triton X-100, 50 mM, Tris-HCl pH 8, 10% glycerol) and incubation at 4°C for 30 min. Purifications were performed using the μMACS™ Epitope Tag Protein Isolation kit (Miltenyi Biotec). Samples containing 7 mg of total protein were incubated with the corresponding magnetic beads-coupled antibody (anti-tag MicroBeads, Miltenyi) at a ratio of 50 μl of antibody per ml and mixtures were incubated for 1 h at 4°C with gentle shaking. Inmunoprecipitations were performed following the instructions provided by the antibody provider. Crosslinking methylene bridges were removed at elution, by incubation for 5 min in a modified SDS-sample buffer (50 mM Tris HCl pH.6.8, 50 mM DTT, 1% SDS, 1 mM EDTA, 0.005% bromphenol blue, 10% glycerol) pre-heated at 95°C. Eluted fractions were resolved by SDS-PAGE in 8–15% polyacrylamide gels.

### Enzymatic assays

Aminoacylation assays were performed as described (Francklyn et al., 2008). Reactions contained 100 mM HEPES [pH 7.2], 20 mM KCl, 30 mM MgCl_2_, 5 mM ATP, 0.1 mg ml^−1^ BSA, 0.5 mM DTT, 20 μM [^14^C]-L-Val (250 Ci mol^−1^), 10 μM tRNA and 5–15 nM enzyme. Reactions were incubated at 30°C and stopped on filters soaked with 5% trichloroacetic acid, washed with 5% trichloroacetic acid and the radioactivity retained was counted by scintillation.

### Cellular ATP content determination

ATP measurements were carried out using a commercial kit (ATP Biomass Kit HS, BioThema) based on luciferase activity following the instructions of the manufacturer. Briefly, 0.1 ng of chlorophyll of a cyanobacterial culture growing in standard conditions (around 25 μl) or incubated for 4 h in the presence of 2.5 μM MSX (L-methionine sulfoximine) was mixed with 50 μl of Extractant B/S and 400 μl of ATP Reagent HS. Light emission was measured in a scintillation counter (LS 6000, Beckman).

### Microscopy

*Anabaena* filaments on solid medium were analyzed by confocal microscopy using a Leica HCX PLAN-APO 63X 1.4 NA oil immersion objective attached to a Leica TCS SP2 confocal laser-scanning microscope. GFP was excited at 488 nm using an argon ion laser. Fluorescent emission was monitored by collection across windows of 500–540 nm (GFP imaging) and 630–700 nm (cyanobacterial autofluorescence).

### Bioinformatics

Cyanobacterial species tree was inferred from concatenated small and large rRNA sequences (Ochoa de Alda et al., 2014). Data set selection, retrieval, concatenation, BMGE trimming (default, PAM100 matrix) and removal of constant sites were performed before bayesian phylogenetic reconstruction. GTR+4Γ+CAT (the evolutionary model that best fits dataset) was selected after carrying out a posterior predictive analysis of different alternatives using the ppred programme implemented in Phylobayes (Lartillot et al., 2009). Phylogenetic reconstruction was achieved using a parallelized version of phylobayes, MPI phylobayes (Lartillot et al., 2013), run at Cipres Gateway High Performance Computing Clusters (Miller et al., 2010). Convergence of two chains was checked with the bpcomp programme, whereby convergence was reached if the maxdiff value of the two chains was < 0.1.

### Protein identification by MS

Protein bands were manually excised from Coomassie-stained gels and subjected to in-gel trypsin digestion (Shevchenko et al., 1996) in a Progest automatic in-gel protein digestor (Genomic Solutions, Cambridgeshire, UK) according to the manufacturer's recommendations for Coomassie-stained samples. To remove dye and SDS impurities, gel plugs were extensively washed with 25 mM ammonium bicarbonate, in-gel reduced with 60 mM dithiothreitol, and S-alkylated with excess iodoacetamide followed by digestion with modified porcine trypsin (Promega, Madison WI) (1:100 wt/wt) at 37°C for 6 h. Peptides were subjected to subsequent extractions in ammonium bicarbonate, 70% acetonitrile, and 1% formic acid, dried down by a Speed-Vac centrifugation and resuspended in 10 μL of 0.1% formic acid.

An Agilent XCT plus ion trap mass spectrometer with a ChipCube interface fed by an Agilent 1100 series nanopump HPLC system was used for MS and MS/MS data acquisition. Samples were concentrated, desalted, and resolved using the ProtID-Chip-150 (II) (Agilent) as described (Martinez-Esteso et al., 2011). MS spectra were scanned at 26,000 m/z per second and MS/MS spectra at 8100 m/z per second in the range 300–2200 m/z. The four most intense precursor ions in MS scans were selected for MS/MS and then passed to an active exclusion list released after 1 min.

Each MS/MS spectra data set (ca. 1200 spectra/run) was processed to determine monoisotopic masses and charge states, to merge MS/MS spectra with the same precursor (Δm/z < 1.4 Da and chromatographic Δt < 15 s) and to select high quality spectra with the Extraction tool of SpectrumMill Proteomics Workbench (Agilent). The reduced data set was searched against the Swissprot forward and reversed protein database without taxonomical restrictions in the identity mode with the MS/MS Search tool of SpectrumMill Proteomics Workbench using the following parameters: trypsin, up to 2 missed cleavages, fixed modification carbamidomethylation of cystein, variable modificacion oxidation of methionine, and a mass tolerance of 2.5 Da for the precursor and 0.7 Da for product ions. Peptide hits were validated first in the peptide mode and then in the protein mode according to the score settings recommended by the manufacturer.

## Results

### Identification of novel aaRSs containing CAAD

**Figure 1:**
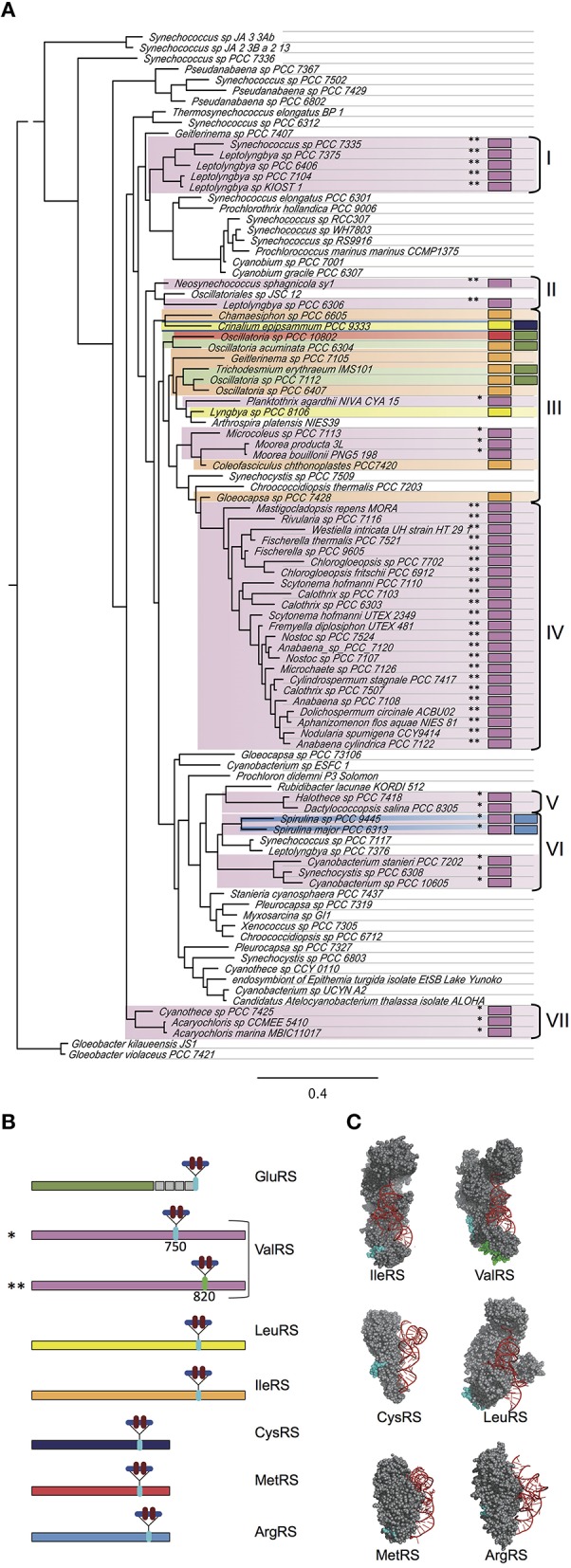
**CAAD in cyanobacterial aaRSs. (A)** Phylogenetic tree of cyanobacterial species based on concatenated small and large rRNA sequences. Representative species of distinct phylogenetic groups were chosen for the construction of the tree. The existence of aaRSs containing CAAD in particular species is indicated with colored boxes following the same color code as in **(B)**. One or two asterisks indicate the insertion of CAAD at positions 750 or 820 in ValRS, respectively. Clades containing species with aaRSs^C^ are indicated by brackets and roman numbers. **(B)** Diagram showing the position of the insertion of CAAD in distinct aaRSs. **(C)** Representation of bacterial aaRSs based on structures in the Protein Data Bank (PDB codes 1FFY, HVS, 2BYT, 1LI5, 2CT8, and 1F7U. The amino acids corresponding to the insertion point of CAAD in cyanobacterial aaRSs are highlighted in cyan. For the ValRS, the insertion points corresponding to positions 750 and 820 are indicated in cyan and green respectively. The diagrams for ValRS, IleRS, and LeuRS have been published elsewhere (Olmedo-Verd et al., 2011) and are shown here for the sake of comparison.

Previous work revealed 14 cyanobacterial species with CAAD inserted in either GluRS, ValRS, IleRS, or LeuRS (Olmedo-Verd et al., 2011). Analysis of newly sequenced genomes evidenced that these CAAD-containing aaRSs (hereafter aaRSs^C^) are widespread in the cyanobacterial radiation. Very interestingly, analysis of new genome sequences also revealed the presence of CAAD in other class I aaRSs, including ArgRS, MetRS and CysRS. Among the 279 sequenced cyanobacteria, 102 contained at least one aaRS^C^. ValRS^C^, the most widespread, was found in 79 species whereas IleRS^C^ was present in 11 species, GluRS^C^ in 5, LeuRS^C^ in 2, ArgRS^C^ in 2, CysRS^C^ in 1 and MetRS^C^ in 1 (Figure 1A, Tables S1–S3). The absence of paralogs in these species indicated that such enzymes are active for tRNA aminoacylation. Eight species contained two aaRS^C^ in four different combinations (LeuRS^C^ + CysRS^C^, ValRS^C^ + ArgRS^C^ GluRS^C^ + MetRS^C^, GluRS^C^ + IleRS^C^). The emerging picture was quite intriguing as two thirds of species contained no aaRSs^*C*^ and one third possessed one or two aaRSs^C^ (Figure 1A). Besides, no organism outside the cyanobacterial phylum was found to contain aaRSs^C^.

In the new aaRSs^C^ detected in this study (ArgRS^C^, MetRS^C^, and CysRS^C^), CAAD is inserted at internal positions C-terminal to the catalytic domain (Figure 1B), which in the 3-D structure correspond to residues of the opposite surface to that interacting with tRNA (Figure 1C), similar to what is observed for ValRS^C^, LeuRS^C^, and IleRS^C^.

In the particular case of ValRS, CAAD was previously found at two alternative insertion points (positions 750 and 820, approximately) in different species, suggesting that each insertion was the result of independent evolutionary events (Olmedo-Verd et al., 2011). In the new species here detected to contain ValRS^C^, CAAD is inserted at either of these positions and species with CAAD at the same position tend to cluster in the phylogenetic species tree (Figure 1A, clusters I to VII), supporting that in clustered species ValRS^C^ is the result of a common insertion event. Phylogenetic analyses of CAAD lend support to this view (Figure S1).

### CAAD functions as a membrane-targeting domain in cyanobacteria and in heterologous systems

**Figure 2:**
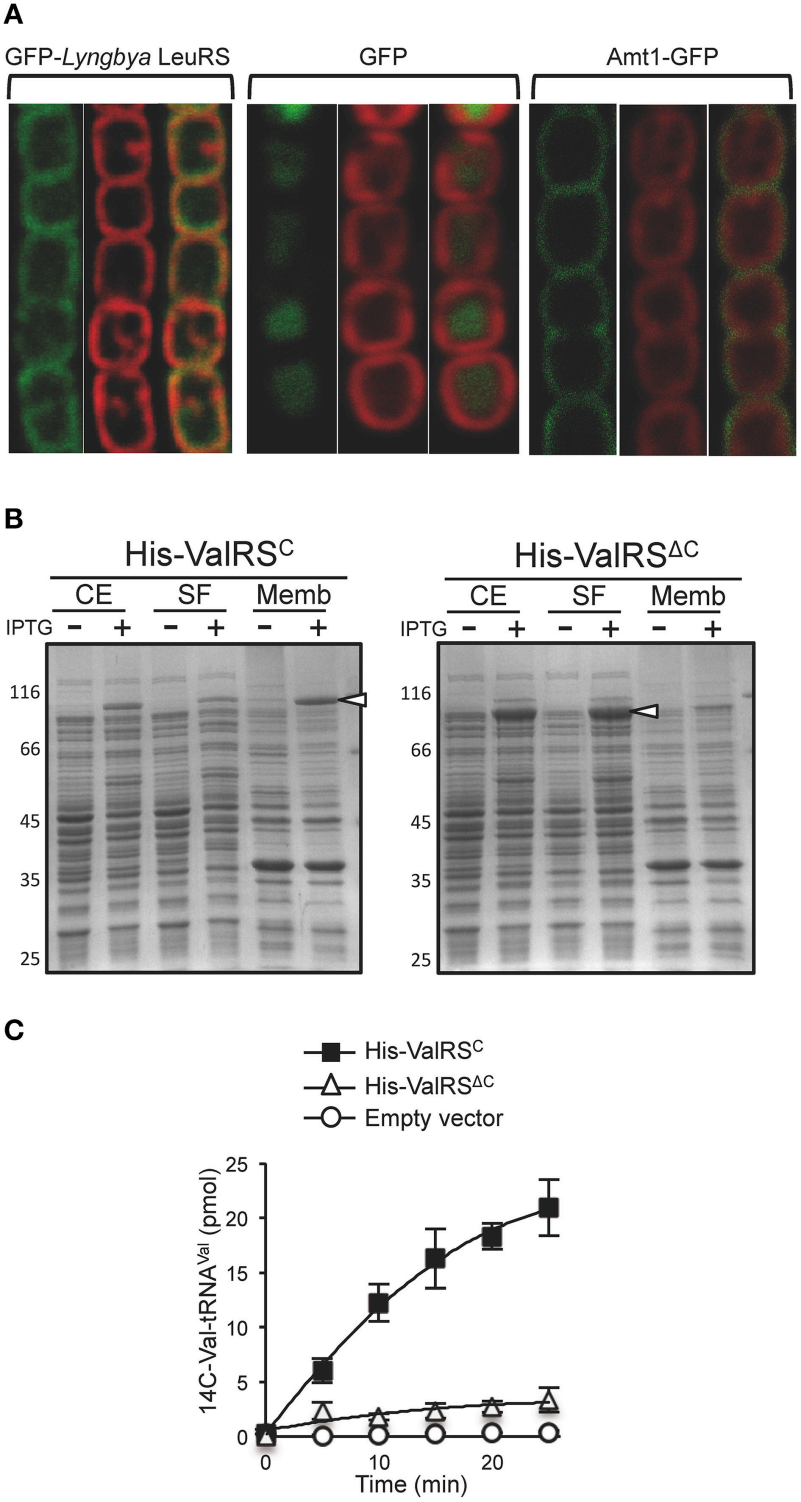
**CAAD possess an inherent membrane targeting ability. (A)** Confocal fluorescent microscopy of *Anabaena* filaments expressing the LeuRS from *Lyngbya* sp. PCC 8106 fused to GFP (left), GFP (center), or the *Anabaena* ammonium transporter Amt1 fused to GFP (right). For each group of pictures, the left panel shows the fluorescence of GFP, the middle panel the red fluorescence of thylakoid membrane pigments and the right panel the merged picture of the former ones. **(B)** SDS-PAGE of fractionated extracts from *E. coli* C41(DE3) expressing *Anabaena* His-ValRS^C^ (left) or His-ValRS^Δ*C*^ (right). CE, whole cell extracts; SF, soluble fraction; MF, membrane fraction. White arrowheads point to the position of the His-ValRS^C^ or His-ValRS^Δ*C*^. **(C)** Aminoacylation assay with membrane fractions from *E. coli* C41(DE3) cells expressing *Anabaena* His-ValRS^C^ or His-ValRS^Δ*C*^. A control of the activity of membranes from *E. coli* C41(DE3) carrying the pET28b vector is shown.

In order to check whether similar to ValRS^C^ in *Anabaena*, other aaRSs^C^ from different species also localize at the thylakoid membrane, genes encoding representatives of each of them were PCR amplified using genomic DNA from the corresponding species, fused to the ORF of the green fluorescent protein (GFP), cloned in a shuttle vector downstream of the *petE* inducible promoter and introduced in *Anabaena* by conjugation. Except for the construction expressing the GFP fusion of LeuRS^C^ from *Lyngbya* sp. PCC 8106, all other constructions showed a strong toxicity in *Anabaena* and ex-conjugants could not be obtained despite multiple attempts, which is in line with previous findings about the barriers to horizontal transfer of genes involved in translation due to toxicity of their gene products (Sorek et al., 2007). For the GFP-LeuRS^C^ fusion, fluorescence of GFP was monitored by confocal microscopy and it was observed to co-localize with the red fluorescence of the photosynthetic pigments, indicating a specific localization in the thylakoid membranes (Figure 2A, left; compare with controls of soluble and plasma membrane proteins in center and right panels). This reproduces the reported localization of the *Anabaena* ValRS^C^-GFP fusion and it is consistent with the observed confinement of tRNA^Leu^ charging activity in membrane fractions of *Lyngbya* cell extracts (Olmedo-Verd et al., 2011).

These observations indicated that similar to the well-characterized ValRS^C^ of *Anabaena*, other CAAD-containing aaRSs from distinct species would also localize in thylakoid membranes and in turn, that CAAD would function as a membrane-targeting domain in different cyanobacterial species. This raised the question of whether CAAD could mediate protein targeting to membranes independently of the host. To test this, the *Anabaena* ValRS^C^ ORF was fused to a His-tag (His-ValRS^C^), expressed in *E. coli* C41(DE3) and its presence was analyzed in sub-cellular fractions. As observed in Figure 2B, His-ValRS^C^ mostly localized in the membrane fraction of cells induced with IPTG. Consistent with this, membrane fractions of these cells exhibited tRNA^Val^ aminoacylation activity, whereas membrane fractions of the recipient *E. coli* strain carrying the empty vector or a plasmid expressing His-ValRS^Δ*C*^ (where CAAD was deleted) were virtually devoid of activity (Figure 2C). ValRS^Δ*C*^ was actually found in the soluble fraction of *E. coli* cells Figure 2B. These results evidenced that CAAD can function as a membrane-targeting domain also in organisms where it does not exist, suggesting that the capacity for membrane targeting is inherent to this domain. It is worth mentioning that *E. coli* does not posses thylakoid membranes, so in this organism CAAD mediates protein targeting to the plasma membrane.

### Subcellular localization of the 20 aaRSs in *Anabaena*

**Figure 3:**
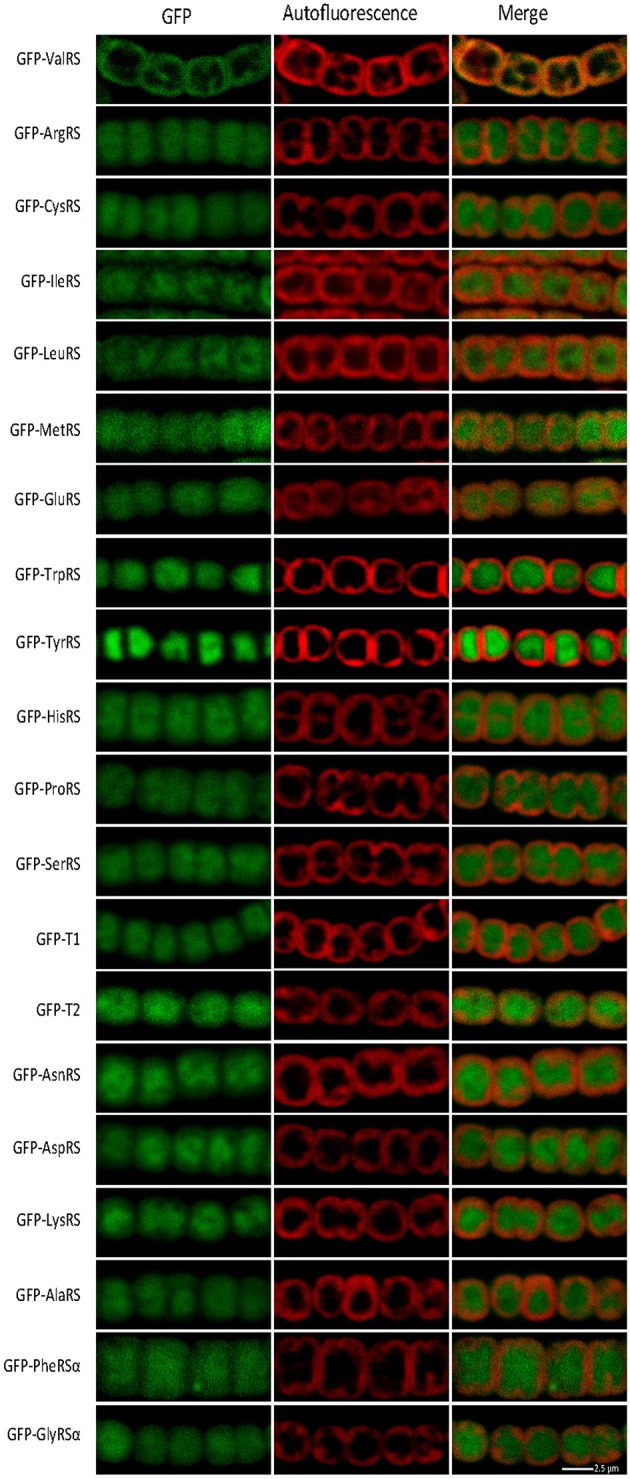
**Confocal fluorescent microscopy of *Anabaena* filaments expressing the indicated fusion protein**. Left panels show the green fluorescence of GFP; central panels, the red fluorescence of photosynthetic pigments and right panels the merged picture of the former ones.

The distribution of aaRSs in cyanobacterial cells was addressed. A possibility to be tested was whether aaRSs not containing CAAD could bind to membranes despite the absence of an apparent membrane-anchoring domain. We hypothesized that, if translation complexes existed, they were likely to accrue around membrane ValRS^C^, as a result of its restricted mobility. To investigate this, *Anabaena* strains were engineered to express each aaRSs fused to GFP from the *petE* inducible promoter and the subcellular localization of the fusion proteins was monitored by confocal fluorescence microscopy. *Anabaena* contains duplicated ThrRSs, named T1 and T2, (Napolitano et al., 2012; Rubio et al., 2015) and similar to many other bacteria, it lacks a GlnRS, Gln-tRNA^Gln^ being synthesized through the indirect pathway (Schon et al., 1986; Luque et al., 2008). For hetero-oligomeric aaRSs, namely GlyRS and PheRS, only one construct fusing the α subunit to GFP was made. Thus, 20 different constructs were introduced in *Anabaena*. Consistently with published data, the GFP-ValRS^C^ fusion showed a sub-cellular distribution that matched the autofluorescence of photosynthetic pigments, used here as an indicator of the position of thylakoid membranes (Figure 3, top panels). By contrast, the fluorescence of all other GFP fusions was detected in the central part of the cytoplasm, with a pattern complementary to that of thylakoid membranes (Figure 3). This signal was similar to that of the soluble GFP protein (Figure 2A) and indicates a sub-cellular distribution of these aaRSs in the soluble portion of the cytoplasm.

In the absence of combined nitrogen, *Anabaena* filaments differentiate specialized cells named heterocysts, which are mostly devoted to nitrogen fixation and show a distinct ultrastructure and morphology. ValRS^C^ was shown to undergo a re-localization along the differentiation process to concentrate at the poles of mature heterocysts (Olmedo-Verd et al., 2011) (see also Figure S2, top panels). The behavior of the 20 GFP-aaRS fusions during differentiation was analyzed here. GFP-aaRSs not containing CAAD did not show any sign of re-localization, so that in mature heterocysts they showed a subcellular distribution similar to that in vegetative cells (Figure S2).

### Complex formation by membrane-bound and soluble aaRSs in *Anabaena*

The asymmetric distribution of aaRSs in *Anabaena*, with 19 soluble and one membrane-bound aaRS was intriguing. We hypothesized that the membrane-bound aaRS could have acquired novel functionality, perhaps not related to its canonical aminoacylation function, that emanates from this new localization. This functionality could be exerted through interaction with other proteins, whose identity could shed light on this issue. On the other hand, soluble aaRSs could also interact with other proteins. To get an insight on this, a global analysis was conducted in search for proteins that interacted with any of the 20 aaRSs in *Anabaena.* The experimental approach consisted in the *in vivo* crosslinking of *Anabaena* cells expressing each GFP-aaRS fusion, followed by cell disruption and purification with anti-GFP antibodies coupled to magnetic beads. For the strains expressing the soluble GFP-aaRSs fusions, proteins were purified from the soluble fraction of cell extracts whereas for the strain expressing ValRS^C^-GFP, purifications were performed from whole cell extracts and membrane fractions (see below). Proteins from each purification were resolved by SDS-PAGE after crosslinking reversal (see Materials and Methods).

**Figure 4:**
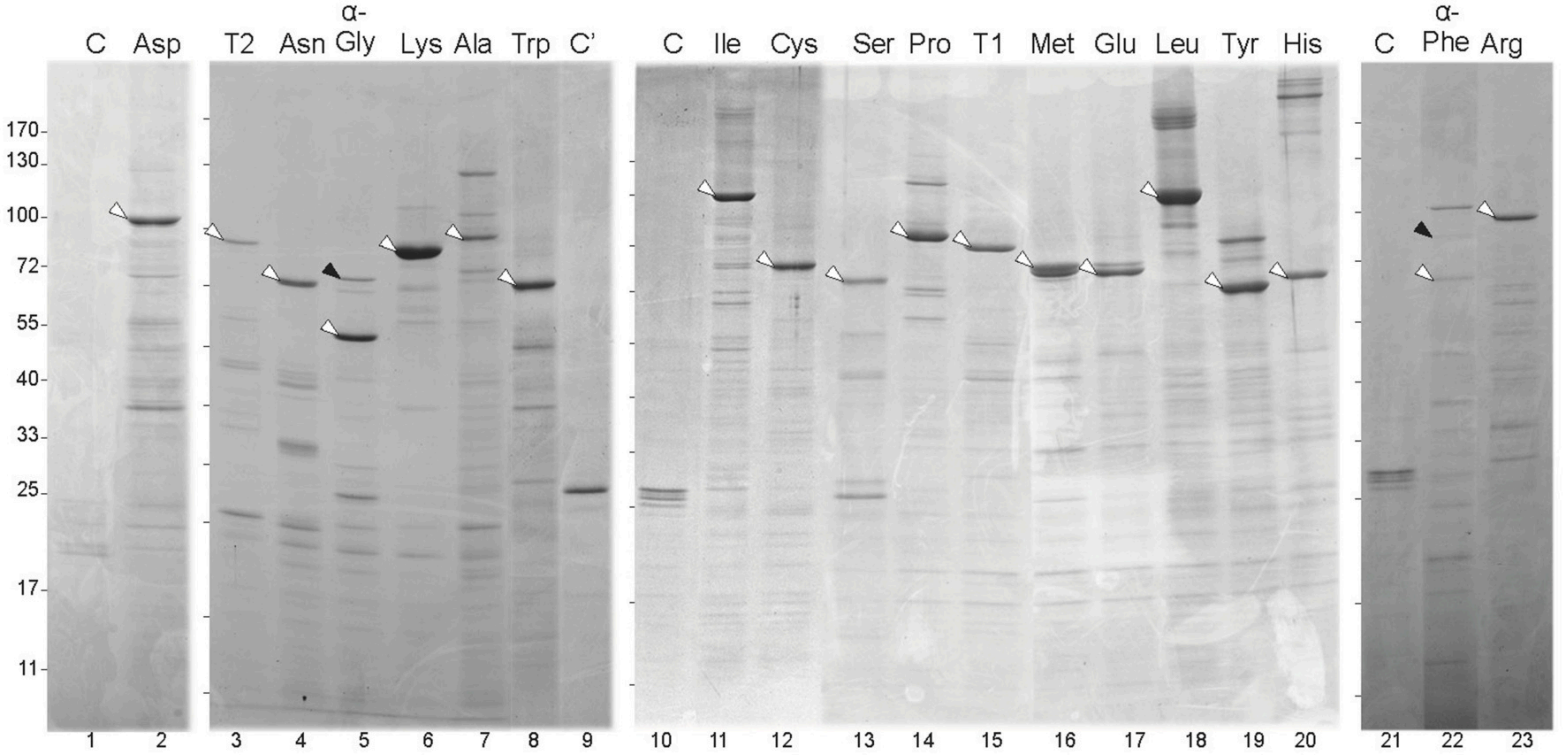
**Purification of GFP-aaRS fusion proteins from *Anabaena* cells**. Panels correspond to SDS-PAGE gels. Each lane contains fractions from the purification of a GFP-aaRS fusion protein (indicated at the top by the cognate amino acid of the aaRS) from *Anabaena* cells expressing it. Control lanes labeled as C or C' carry purification fractions of *Anabaena* cells or *Anabaena* cells expressing GFP, respectively. White arrowheads point to the corresponding fusion protein. Black arrowheads point at the beta subunit of GlyRS (lane 5) and PheRS (lane 22). Marks at the left side of each panel indicate the position of the bands of the PageRuler™ Prestained Protein Ladder (Fermentas).

For each of the soluble aaRSs, a major band coincident with the expected MW of the respective GFP fusion was observed in SDS-PAGE gels (Figure 4, white arrowheads) and both GFP and the corresponding aaRS were identified in such bands by mass spectrometry (MS). Differential bands (i.e., those that were absent from control lanes containing proteins purified from *Anabaena* wild-type cells or a strain expressing GFP) that putatively corresponded to proteins interacting with aaRSs, were selected for MS identification. Quite surprisingly, most of the 141 bands subjected to MS identification corresponded to putative proteolytic products of the corresponding aaRS (despite the rapid manipulation of samples at low temperature and the regular addition of a cocktail of protease inhibitors to all solutions). However, some proteins that corresponded to putative aaRS interactants were identified and are listed in Table S4. It is crucial to mention that GFP-α-GlyRS and GFP-α-PheRS co-purified with their respective β subunits (Figure 4, lanes 5 and 22, black arrowheads), which validated this approach at least for the co-purification of proteins forming stable complexes.

**Figure 5:**
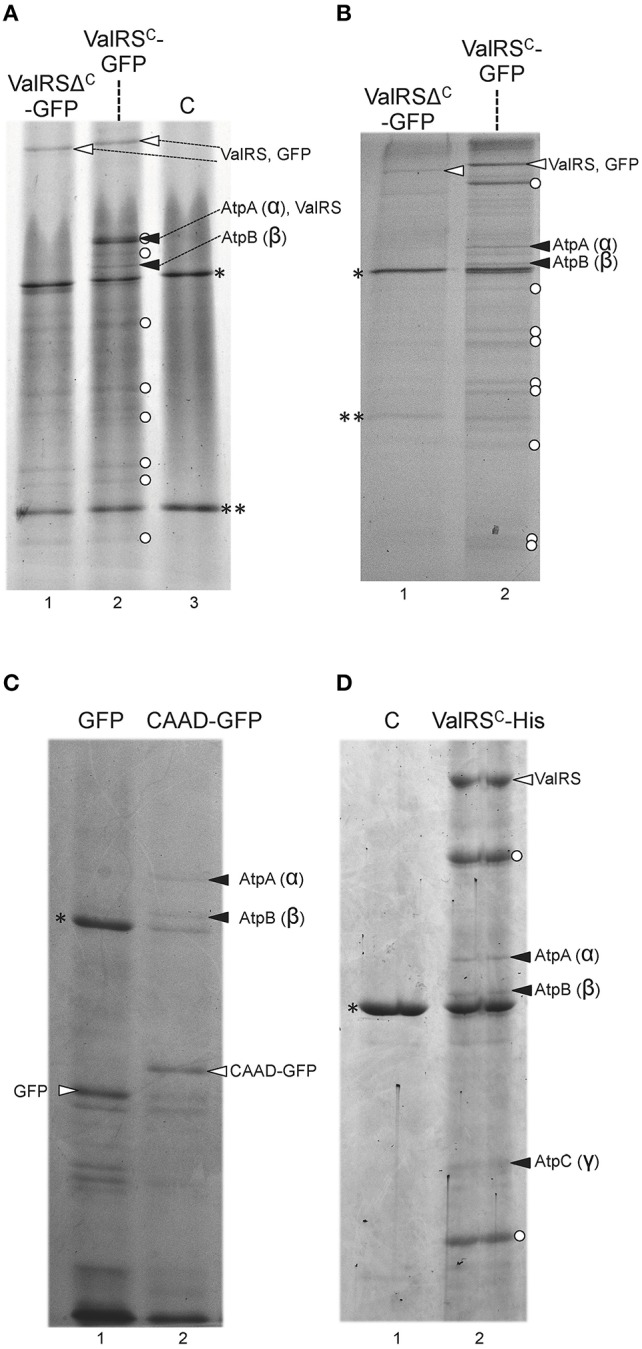
**Co-purification of *Anabaena* ValRS^C^ with subunits of the ATP synthase. (A)** Whole cell extracts from *Anabaena* strains expressing the fusion protein indicated at the top of the panel were purified using anti-GFP antibodies and resolved by SDS-PAGE gel. Control lane labeled as “C” contains purification fractions of *Anabaena* cells not expressing any fusion protein. White arrowheads point to the corresponding fusion protein. Black arrowhead point to subunits of ATP synthase. White dots indicate proteolytic fragments of the GFP fusion proteins. Asterisks indicate the position of RubisCO, double asterisks indicate the position of the light chain of inmunoglobulins **(B)** and **(C)** Membrane fractions of *Anabaena* strains expressing the fusion protein indicated at the top of the panel were purified using anti-GFP antibodies and resolved by SDS-PAGE gel. Other details are like in (**A)**. **(D)** Membrane fractions of *Anabaena* strains expressing the fusion protein indicated at the top of the panel were purified using anti-hexahistidine antibodies and resolved by SDS-PAGE gel.

In the particular case of ValRS^C^-GFP, numerous bands corresponding to putative proteolytic products (labeled with white dots in Figure 5) were also observed in the electrophoreses, despite measures mentioned above were taken to avoid proteolysis. ValRS^C^-GFP was readily detected in purifications from whole cell extracts (Figure 5A, white arrowheads). Interestingly, specific bands (Figure 5A, black arrowheads), observed in the lane containing ValRS^C^-GFP that were absent in lanes containing fractions from control strains expressing ValRS^Δ*C*^-GFP or no fusion protein (Figure 5A, lanes 1 and 3, respectively) were identified as subunits α (AtpA) and β (AtpB) of the FoF1-ATP synthase. Further purifications were conducted using membrane fractions of the above-mentioned strains and again, subunits α (AtpA), β (AtpB) and, in some experiments, subunit γ (AtpC) of the ATP synthase complex were detected to co-purify specifically with ValRS^C^-GFP (Figure 5B, black arrowheads). Importantly, subunits of the ATP synthase did not co-purify with GFP-ValRS^Δ*C*^ from either whole-cell extracts (Figure 5A, lane 1) or membrane fractions (Figure 5B, lane 1), suggesting that CAAD would be directly involved in the interaction. To test this, membrane fractions from *Anabaena* strains engineered to express GFP-CAAD or GFP were used for purification experiments similar to those described above. Two bands identified as AtpA (α) and AtpB (β) were detected in the lane corresponding to the strain expressing GFP-CAAD (Figure 5C, lane 2) but not in the control lane corresponding to that expressing GFP (Figure 5C, lane 1), indicating that CAAD is directly involved in the interaction with these proteins.

To rule out artifacts derived from the use of the GFP tag in the experiments above, membrane fractions of a strain engineered to express ValRS^C^ fused to a C-terminal His tag were used for purification with anti-His tag antibodies coupled to magnetic beads after *in vivo* cross-linking. Proteins that co-purified specifically with ValRS^*C*^-His were identified as AtpA (α), AtpB (β), and AtpC (γ) (Figure 5D, lane 2).

**Figure 6:**
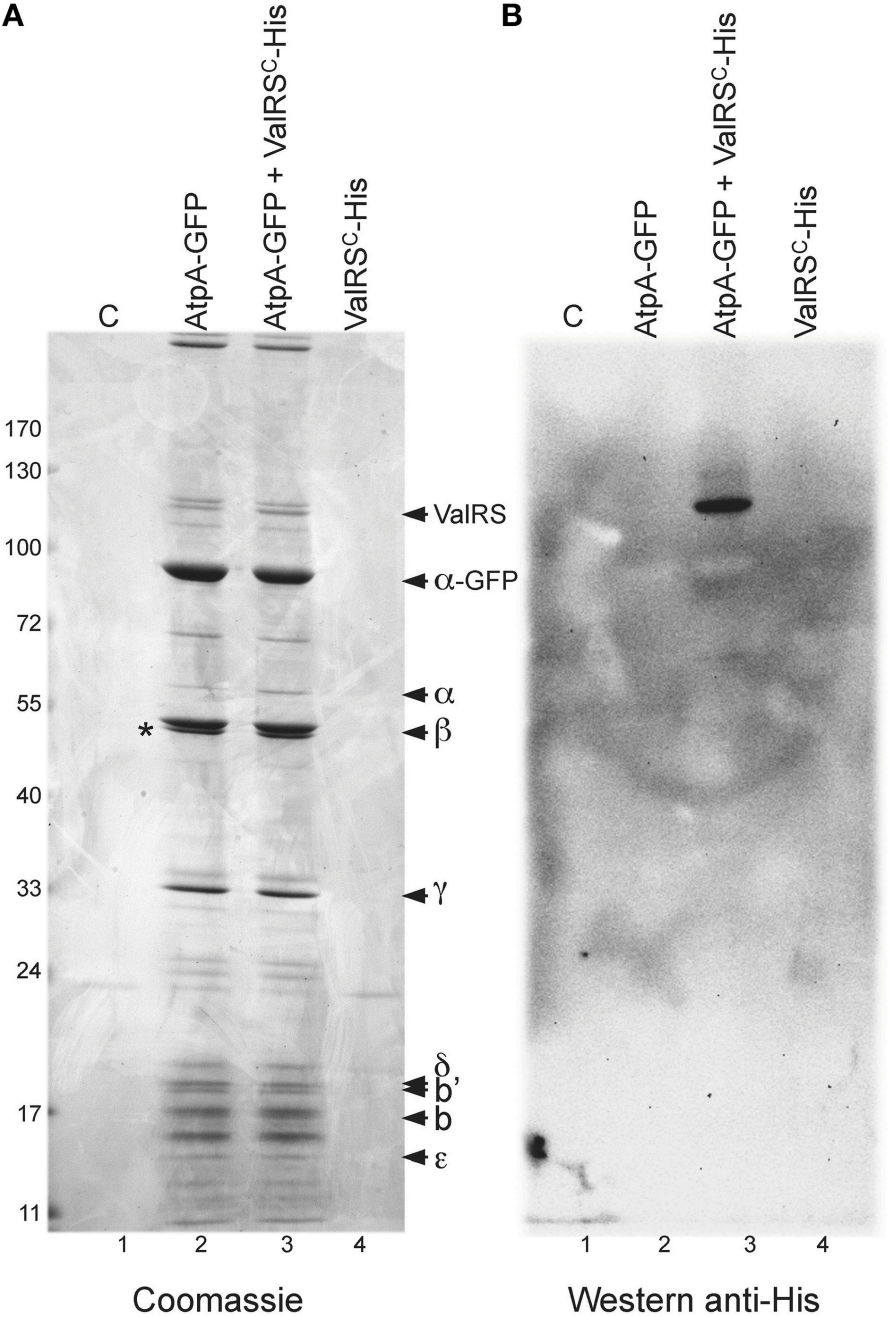
**Co-purification of *Anabaena* AtpA-GFP with other subunits of the ATP synthase and ValRS^C^ or ValRS^C^-His**. **(A: Left)**, SDS-PAGE gel containing fractions purified with anti GFP-antibodies coupled to magnetic beads. Lanes contain purification fractions from *Anabaena* cells expressing the proteins indicated at the top. Control lane labeled as C carry purification fractions from *Anabaena* cells expressing no fusion protein. Proteins identified by mass spectrometry are indicated. An asterisk indicates the position of RuBisCO. **(B: Right)**, western blot of a gel identical to that of the left panel using an antibody against hexahistidine tag.

Further evidence for this interaction was sought by reciprocal co-purification experiments. The *atpA* ORF, encoding the α subunit of the ATP synthase, was fused to the ORF encoding GFP and the resulting construct was introduced in wild-type *Anabaena* and in the strain expressing ValRS^C^-His. Cultures of these strains and control strains were subjected to *in vivo* crosslinking and purification with anti-GFP antibodies coupled to magnetic beads. Purified fractions were resolved by SDS-PAGE after crosslinking reversal (Figure 6A). A number of bands were observed in lanes corresponding to the strains expressing AtpA-GFP in a wild-type background (lane 2) or a background expressing ValRS^C^-His (lane 3), but not in lanes 1 and 4 corresponding to control strains not expressing AtpA-GFP (lane 1, wild-type background; lane 4, ValRS^C^-His background). AtpA-GFP was identified as the most intense band in lanes 2 and 3 by size and MS. Other prominent bands contained subunits β, γ, δ, b, b′, and ε of the ATP synthase. Importantly, the intensity of the bands was consistent with the subunit stoichiometry of ATP synthase, indicating that AtpA-GFP had successfully integrated in the FoF1 complex. The presence of a minor band matching the size and identified by MS as the endogenous α subunit (AtpA), indicated that some of the complexes contained both AtpA-GFP and AtpA subunits. Crucially, a specific band with a molecular weight of ca. 110 kDa only present in lanes 2 and 3 was identified by MS as ValRS^C^. A band of similar size was revealed by western blot with anti-His antibodies (Figure 6B, lane 3), corroborating the presence of ValRS^C^-His at this position.

**Figure 7:**
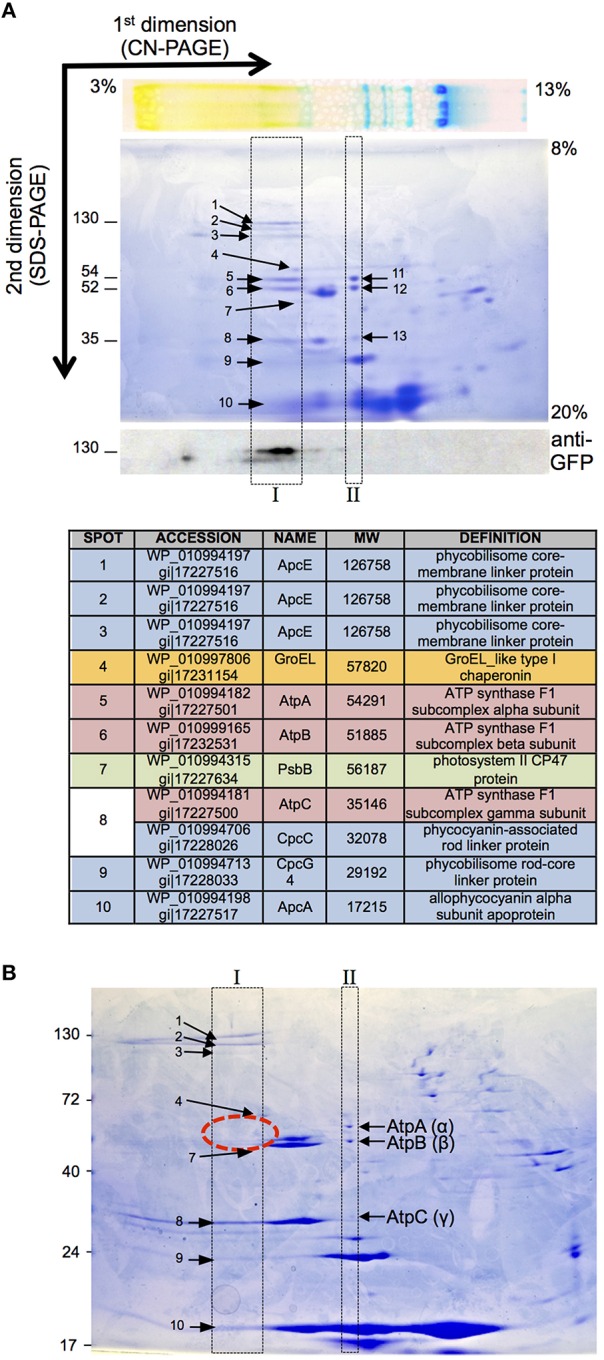
**CN_PAGE of membrane fractions of *Anabaena* strains. (A)** CN-PAGE of a membrane preparation of *Anabaena* cells expressing ValRS^C^-GFP. The top panel shows a picture of the sample separated in the first dimension previous to the separation in the second dimension. The bottom panel shows a western blot of a gel identical to that of the central panel using an antibody against GFP. Boxes indicate the vertical lines where ATP synthase is found in the CN-PAGE gel. Numbers refer to proteins identified by mass spectrometry that are listed in the table. **(B)** CN-PAGE of a membrane preparation of the *Anabaena* Δ^C^ mutant.

Results above strongly indicate that ValRS^C^ interacts with the ATP synthase complex in the thylakoid membranes of *Anabaena*. A technique extensively used for the identification and analysis of membrane protein complexes, including photosynthetic and respiratory complexes, is 2D-native electrophoresis that includes blue-native and colorless-native electrophoresis. In these gels, protein complexes are resolved in the first dimension in native conditions (in the presence of a mild detergent) and after denaturation, the components of each complex are separated in the second dimension by SDS-PAGE. Thus, in 2D-native gels the components of a particular protein complex distribute within a vertical line. To analyze complex formation by ValRS^C^ in *Anabaena*, colorless-native (CN-native) electrophoresis was chosen for its better preservation of labile complexes (Wittig et al., 2007). To facilitate detection, membrane preparations of the *Anabaena* strain expressing ValRS^C^-GFP were utilized, so that its position in the gels could be ascertained by western blot with anti-GFP antibodies. ValRS^C^-GFP, was detected in the first dimension in the high-molecular-weight zone of the gel, suggesting its integration in a large complex. In the second dimension, it occupied a position consistent with its expected molecular weight (130 kDa; Figure 7A, bottom panel). Proteins in the same vertical line as ValRS^*C*^-GFP (Figure 7A, box I) were identified by MS and are listed in Figure 7A. Very importantly, AtpA, AtpB, and AtpC were among them (Figure 7A spots 5, 6 and 8). AtpA, AtpB, and AtpC were also identified in a vertical line elsewhere in the gel, at a position that corresponded to a complex of a smaller size (Figure 7A, spots 11, 12, and 13 in box II). These observations suggested that part of the ATP synthase complex would be shifted in the gel to a position of a higher molecular weight by interaction with ValRS^C^-GFP. When CN-native gels were made with membrane preparations of a mutant named Δ^C^, where CAAD was deleted from ValRS (see below), ATP synthase subunits were only observed at the low molecular weight complex (Figure 7B, box II), indicating that their presence in the high molecular weight complex (box I) was dependent on the presence of ValRS^C^ in the membranes.

**Figure 8:**
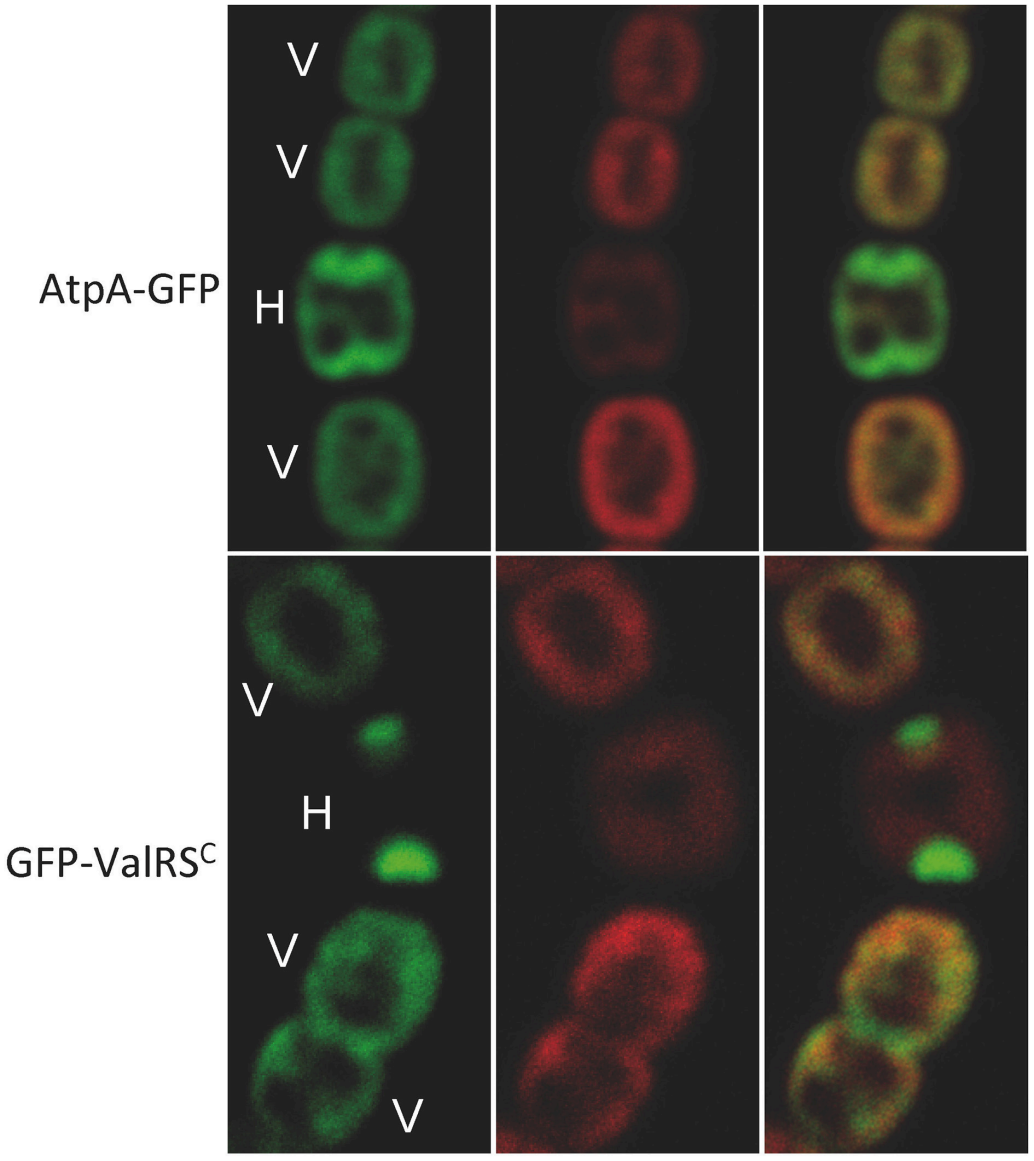
**Subcellular localization of AtpA**. Pictures correspond to confocal microscopy images of *Anabaena* cells expressing the protein indicated at the left margin. Left panels show the green fluorescence of GFP, central panels the red fluorescence of photosynthetic pigments and the right panel the merged picture of the former ones. V, vegetative cells; H, heterocyst.

The subcellular localization of the FoF1-ATP synthase in *Anabaena* was examined by monitoring the fluorescence of the AtpA-GFP fusion. As observed in Figure 8, the green fluorescence of AtpA-GFP in vegetative cells co-localized with the red auto-fluorescence of photosynthetic pigments indicating localization of the ATP synthase complex in the thylakoid membranes, which is similar to the distribution of ValRS^C^-GFP. In mature heterocysts, where ValRS^C^ concentrates at the cell poles, the distribution of AtpA-GFP overlapped but was not totally coincident with that of GFP-ValRS^C^, showing also some enrichment at the cell poles.

### Functional consequences of the ValRS-ATP synthase interaction

Given the difficulty to reconstitute the interaction between ValRS and ATP synthase *in vitro*, we analyzed the impact of the interaction on the activity of ValRS by measuring aminoacylation activity in whole cell extracts of the wild type strain and the *Anabaena* Δ^C^ mutant, where the interaction between ValRS and ATP synthase was not expected to occur. The only genetic modification of this mutant was the replacement of the *valS* gene by a mutant alelle encoding the ValRS^Δ*C*^ protein, not altering the *valS* promoter or any other sequence in the genome. It is worth to note that at difference with the WT strain, the tRNA^*Val*^ aminoacylation activity was found in the soluble fraction in this mutant (Figure S3A). Whole cell extracts of the WT strain and the Δ^C^ mutant showed similar tRNA^Val^ aminoacylation activity [0.115 ± 0.009 and 0.128 ± 0.010 pmol (^14^C)-Val-tRNA^Val^ min^−1^ · μg protein^−1^, respectively (numbers are the mean aminoacylation activity of three independent cultures±standard deviation)] suggesting that the interaction with ATP synthase does not have a strong impact on the activity of the aaRS. The alternative hypothesis that the interaction would have an impact on the activity of the FoF1 complex was considered. To test this, the ATP content of cells was measured as a proxy of the activity of the FoF1 complex. Wild type and Δ^C^ cells had similar ATP content [2.01 mM ± 0.12 and 1.87 mM ± 0.33, respectively (numbers are the mean ATP concentration of three independent cultures±standard deviation)]. Since it was previously observed that an *Anabaena* mutant where CAAD had been deleted from ValRS^C^ (similar to the Δ^C^ mutant) was impaired for growth upon nitrogen depletion (induced by treatment with L-methionine sulfoximine, MSX), we assessed the ATP content of wild type and Δ^C^ cells growing in the absence or presence of MSX. No significant differences were observed between wild type or Δ^C^ cells growing under standard or nitrogen-depleted conditions (Figure S3B).

## Discussion

### Recurrent acquisition of CAAD by cyanobacterial aaRSs in evolution

Previous work demonstrated the existence of CAAD in four class I aaRS, namely GluRS, LeuRS, ValRS, and IleRS in different cyanobacterial species (Luque et al., 2008; Olmedo-Verd et al., 2011). The fast accumulation of genome sequences in recent years has allowed the unveiling of new species bearing these aaRSs^C^ and species where CAAD is inserted in additional aaRSs, namely ArgRS, MetRS, and CysRS. Thus, as more genomes are sequenced, the variety of aaRSs containing CAAD increases, raising the question of whether there exist other aaRSs^C^ that are still to be discovered. A common feature of aaRSs^C^ identified thus far is that they belong to class I and they are all monomeric. AaRSs not observed to contain CAAD are either of class I (TyrRS and TrpRS) or class II, and are all dimeric or tetrameric. It is possible that for these oligomeric aaRSs, anchoring of each individual subunit to the membrane would posit structural constrains for the assembly of the functional oligomer, making less likely the recruitment of CAAD by these enzymes.

CAAD most likely originated by *domain shuffling* (Di Roberto and Peisajovich, 2014), a loosely defined phenomenon by which genes acquire or lose sequences encoding individual modules. In the case of GluRS, CAAD acquisition probably happened by fusion of neighboring genes, which is consistent with the observation of contiguous *gltX* and *curt1* genes in species closely-related to those containing GluRS^C^ (i.e., *Lyngbya* sp PCC 8106, *Arthrospira* sp. PCC 8005, *Arthrospira maxima* CS-328 or *Oscillatoria* sp. PCC 6506). It is unknown whether insertion of CAAD in other aaRSs^C^ occurred by recombination, transposition or an alternative mechanism. If CAAD were a sequence that inserts into new locations randomly, footprints would be expected at multiple sites in the genome. Rather than this, specific recruitment by aaRSs is observed. Phylogenetic analysis suggests that at certain points of cyanobacterial evolution, aaRSs-encoding genes independently captured the sequence of a *curt1* gene, which would involve a minimum of eight independent recruitment events, one for each aaRS^C^, except for ValRS where CAAD is found at two alternative insertion points. However, phylogenetic analyses of the Curt1/CAAD superfamily indicate multiple recruitment events by ValRS^C^, IleRS^C^, and LeuRS^C^ (Figure S1). Some events are recent and the resulting aaRSs^C^ are restricted to few species (Figure 1, cluster III), whereas others are ancient and can be traced to billion years ago (Figure 1, clusters IV to VII) (Knoll, 2008; Schirrmeister et al., 2013). A striking and interesting observation is the clustering of CAAD sequences from distinct aaRSs^C^ in the phylogenetic tree in Figure S1 (clusters labeled as III), which suggests that some aaRSs^C^ might have acquired CAAD from another aaRS^C^, a possibility that warrants further investigation.

The recurrent recruitment of CAAD by distinct aaRSs in cyanobacteria strongly suggests a putative functionality of aaRSs^C^ in the thylakoid membrane. It is thus intriguing that aaRSs^C^ do not exist in plant chloroplasts, which derive from ancient cyanobacteria and contain CURT1 proteins. This may be due to the fact that the cyanobacterial ancestor of chloroplasts did not contain any aaRS^C^ and posterior recruitment events have not taken place in the evolution of chloroplasts. It should be also noticed that in *Arabidopsis thaliana*, only nine of the plastidic aaRSs (including GluRS and IleRS) are of cyanobacterial origin, the others having been replaced by aaRSs of cytoplasmic or mitochondrial origin. Targeting of aaRSs (including plastidic GluRS and IleRS) to multiple compartments in the cell is the general rule in plants (Duchene et al., 2005), which may have disfavor the existence of aaRSs^C^ in plant plastids.

### A global picture of the subcellular distribution of aaRSs in cyanobacterial cells

Though it was known that in *Anabaena* ValRS^C^ localized specifically at thylakoid membranes (Olmedo-Verd et al., 2011), it remained to be investigated whether other aaRSs^C^ also localized in the thylakoids. Our attempt to ectopically express foreign aaRSs^C^ in *Anabaena* as GFP fusions was hampered by the high toxicity of these enzymes in the host, even when expressed from a controlled promoter. Only LeuRS^C^ from *Lyngbya* sp. PCC 8106 was successfully expressed and was found to co-localize with photosynthetic pigments at the thylakoids (Figure 2A). This is consistent with the previous observation of LeuRS activity in membrane fractions from *Lyngbya* (Olmedo-Verd et al., 2011) and argues in favor of the localization of all aaRSs^C^ at the thylakoid membranes of the corresponding species. Consistent with this view is the intrinsic membrane-targeting ability of CAAD observed in cyanobacteria (Olmedo-Verd et al., 2011) and *E. coli* (Figure 2B). The inherent ability of CAAD to insert in a lipid bilayer (i.e., the plasma membrane of *E. coli*) raises the question of how in cyanobacteria it specifically inserts in thylakoid membranes. Plausible hypotheses are the existence of a cue that attracts CAAD for specific insertion in thylakoids or the existence of an interacting partner that captures CAAD in these membranes. Either role could be played by the interaction with ATP synthase described in this work.

The information available on the subcellular localization of aaRSs lacking CAAD was scanty. In this work we provide a global view for the subcellular distribution of the full complement of aaRSs in *Anabaena*. Analysis of GFP fusions for all 20 aaRSs indicated that with the exception of ValRS^C^, all other aaRSs are soluble. Though this is not in line with our hypothesis that ValRS^C^ could attract other aaRSs for the formation of translational foci on the surface of thylakoids, we cannot rule out that these foci formed only under specific conditions. In this sense it is worth mentioning that in *Synechocystis* sp. PCC 6803, components of the translational machinery, inlcuding β-GlyRS, were observed to form aggregates on the surface of thylakoid and plasma membranes under high light stress (Bryan et al., 2014).

### Identification of aaRSs interacting partners

The asymmetric subcellular distribution of aaRSs in many cyanobacterial species is intriguing. To get an insight on the basis for this differential distribution, interactions of each aaRS with other proteins were investigated in *Anabaena*. A number of soluble aaRSs, including IleRS, CysRS, TyrRS, TrpRS, AlaRS, HisRS, ProRS, and AsnRS appeared not to form stable complexes. By contrast, indications of putative interactions of LeuRS, ArgRS, MetRS, GluRS, T1, SerRS, LysRS, and AspRS with other proteins were obtained (Table S4). These putative interactions would need to be validated by alternative approaches and further investigated. Particularly interesting are the observations indicating a possible interaction of SerRS, LeuRS, MetRS, and T1 with EF-Tu. Since co-purification of these aaRSs with each other was not observed, it appears that EF-Tu would establish binary interactions with each of them rather than acting as a hub for the formation of a large multi-synthetase complex. Several aaRSs have been reported to interact with EF-Tu in bacteria or with its homolog EF1A in archaea or eukaryotes (Motorin et al., 1991; Bec et al., 1994; Reed et al., 1994; Negrutskii et al., 1999; Hausmann et al., 2007; Guzzo and Yang, 2008). AaRSs and elongation factor Tu and 1A perform successive steps in translation: the aminoacylation of tRNA and the delivery of the aminoacyl-tRNA to the ribosome, respectively. Therefore, the interaction of aaRSs with EF-Tu or EF1A is thought to favor the transfer of aminoacyl-tRNAs to the ribosome. Direct association of aaRSs with ribosomes has been observed in archaea (Godinic-Mikulcic et al., 2011; Raina et al., 2012) and eukaryotes (David et al., 2011), lending support to the idea that the channeling of aminoacyl-tRNAs to the ribosome is advantageous (Negrutskii and Deutscher, 1991; Stapulionis and Deutscher, 1995; Kyriacou and Deutscher, 2008; Mirande, 2010).

AaRSs frequently form protein complexes in archaea and eukaryotes, while in bacteria it is less common (Hausmann and Ibba, 2008). Some protein interactions contribute to the canonical function of aaRSs in translation, either by facilitating the contact with the tRNA substrate, by enhancing the aminoacylation activity or by favoring substrate channeling (Motorin et al., 1991; Bec et al., 1994; Reed et al., 1994; Simos et al., 1996; Negrutskii et al., 1999; Rocak et al., 2002; Praetorius-Ibba et al., 2005, 2007; Godinic et al., 2007; Hausmann et al., 2007; Guzzo and Yang, 2008; Godinic-Mikulcic et al., 2011). Besides, numerous aaRSs are moonlighting proteins with alternative functions different from their canonical role in gene translation and performing such non-canonical functions often entails interactions with alternative partners distinct from their regular partners in translation. Such interactions may occur through extra domains appended to the conserved body of the aaRS (Sampath et al., 2004; Guo et al., 2010; Kim et al., 2012; Ofir-Birin et al., 2013).

In this work, the interaction of ValRS^C^ with the FoF1 ATP synthase complex was unveiled. Such interaction was repeatedly observed by multiple alternative approaches. Purification of ValRS^C^-GFP from whole cell extracts (Figure 5A) or membrane fractions (Figure 5B) repetitively pulled out subunits α (AtpA), β (AtpB) and, in some cases, subunit γ (AtpC) of the FoF1-ATP synthase complex. Artifacts derived from the use of particular tags were ruled out (Figures 5A–D). However, it could be argued that similar to RuBisCO, a frequent contaminant in our purifications (indicated by an asterisk in Figures 5, 6), ATP synthase subunits appeared in our gels just because of their abundance. This possibility was discarded by reciprocal co-purification of ValRS^C^ with GFP-tagged AtpA. In *Anabaena*, AtpA-GFP showed a thylakoidal localization and co-purified with most subunits of the ATP synthase, indicating an effective integration in the FoF1 complex. Further support for the proposed interaction between ValRS^C^ and ATP synthase was derived from CN-PAGE experiments. In these gels, ATP synthase subunits were observed in two complexes (Figure 7) and its presence in the high molecular weight complex was dependent on the presence of ValRS^C^ in the membrane. Both ValRS^C^-GFP and AtpA-GFP localized in thylakoid membranes in vegetative cells, however, in heterocysts they showed an overlapping though not totally coincident distribution enriched at the cell poles indicating that in these cells their interaction could be restricted to the cell poles (Figure 8).

### An insight on the function of the ValRS^C^-ATP synthase interaction

The functional consequences of the interaction of ValRS^C^ with ATP synthase were investigated. Interaction with ATP synthase occurs through CAAD (Figure 5C) and deletion of such domain in the Δ^C^ mutant has two consequences, the disruption of the interaction with ATP synthase and the conversion of the synthetase to a soluble form in the cytoplasm. However, deletion of CAAD does not alter the catalytic parameters (*K*_*M*_ for tRNA, L-Val or ATP and *k*_*cat*_) of the enzyme (Olmedo-Verd et al., 2011). The interaction was deduced to have little impact on ValRS^C^ catalysis as wild type cells and cells of the Δ^*C*^ mutant showed similar tRNA^Val^ charging activity (Figure S3A). Thus, the alternative hypothesis that the interaction would affect ATP synthase functioning was considered. The FoF1 complex possesses both ATP synthase and ATP hydrolase activity, and both need to be finely tuned to the cell status (Gledhill et al., 2007; Giorgio et al., 2009; Hisabori et al., 2013). In photosynthetic organisms, it is well-known that the functioning of the FoF1 complex is tightly regulated by redox and metabolic stimuli, which is required for optimal performance and adaptation to light conditions (Konno et al., 2006; Imashimizu et al., 2011; Kramer and Evans, 2011; Hisabori et al., 2013; Kohzuma et al., 2013). ATP levels were used as an indicator of the functioning of the FoF1 complex and were found to be similar in wild type and Δ^C^ cells. Given that we had previously observed retarded growth in an *Anabaena* mutant lacking the CAAD domain of ValRS (similar to the Δ^C^ mutant) in nitrogen depleted conditions, we hypothesized that ValRS could monitor the nitrogen status by acting as a sensor of amino acid levels (i.e., by sensing valine) and transduce this signal to the FoF1 complex in order to adjust its functioning. Such signaling role would be analogous to that recently proposed for LeuRS in yeasts and mammals (Bonfils et al., 2012; Han et al., 2012). This hypothesis was particularly attractive since in principle, any aaRS could act as an amino acid sensor and therefore, provided that the variety of aaRSs^C^ present in other species interact with the FoF1 complex, they could each play the same role in their host by sensing their cognate amino acid and transducing such information. However, no significant differences in the ATP levels were observed between wild type *Anabaena* or Δ^C^ cells under standard or nitrogen depleted conditions (Figure S3B). Though these results do not confirm our hypothesis, it is possible that the impact of the interaction on the functioning of the FoF1 complex is subtle and would not be reflected in the ATP content of the cell. Therefore the functional consequences of the interaction would need to be further investigated by detailed analyses of the ATP synthase and hydrolase activity of the FoF1 complex.

Membrane localization is unusual for aaRSs. Membrane-associated aaRSs have been described in other organisms (Castro De Moura et al., 2011; Kim et al., 2012) but only in cyanobacteria there exist permanently-anchored and catalytically-active aaRSs in the thylakoid membrane. The observed interaction of ValRS^C^ with the FoF1 ATP synthase complex unveiled in this work hints to a possible moonlighting function of ValRS^C^, which would need to be further investigated. Elucidation of this putative role would help understand the basis for the asymmetric distribution of aaRSs observed in these organisms. We do not rule out however, the possibility that ATP synthase plays a moonlighting role in the context of the complex with ValRS.

## Author contributions

JO and IL conceived and designed research; JS, EO, RB, and IL performed experiments; all authors analyzed and interpreted the data and IL wrote the manuscript

## Funding

This work was supported by grants BFU2010-19544 and BFU2013-44686-P from Ministerio de Economía y Competitividad of Spain and FEDER.

### Conflict of interest statement

The authors declare that the research was conducted in the absence of any commercial or financial relationships that could be construed as a potential conflict of interest.
