# Supplementary material for: Sub-Cellular Localization and Complex Formation by Aminoacyl-tRNA Synthetases in Cyanobacteria: Evidence for Interaction of Membrane-Anchored ValRS with ATP Synthase
Source: Front Microbiol. 2016 Jun 6;7:857. doi: 10.3389/fmicb.2016.00857 (PMC4893482; doi:10.3389/fmicb.2016.00857)
Supplement: Supplementary file 1 [file Table1.PDF]

Table S1. Cyanobacterial genomes containing genes encoding aaRSs[C]

Gene ID	Gene Product Name	Genome
2506598597	arginyl-tRNA synthetase (EC 6.1.1.19)	*Spirulina subsalsa PCC 9445*
2506610414	arginyl-tRNA synthetase (EC 6.1.1.19)	*Spirulina major PCC 6313*
2504684340	cysteinyl-tRNA synthetase (EC 6.1.1.16)	*Crinalium epipsammum PCC 9333*
638105535	glutamate--tRNA(Gln) ligase (EC 6.1.1.24)/glutamyl-tRNA synthetase (EC 6.1.1.17)	*Trichodesmium erythraeum IMS101*
2504092697	glutamyl-tRNA synthetase	*Oscillatoria nigro-viridis PCC 7112*
2505170766	glutamyl-tRNA synthetase, bacterial family	*Microcoleus vaginatus PCC 9802*
2509421051	glutamyl-tRNA synthetase, bacterial family	*Oscillatoria acuminata PCC 6304*
2509512529	glutamyl-tRNA synthetase, bacterial family	*Oscillatoria sp. PCC 10802*
638108551	Isoleucyl-tRNA synthetase (EC 6.1.1.5)	*Trichodesmium erythraeum IMS101*
647564989	Isoleucyl-tRNA synthetase (EC 6.1.1.5)	*Microcoleus chthonoplastes PCC 7420*
648859603	Isoleucyl-tRNA synthetase (EC 6.1.1.5)	*Oscillatoria sp. PCC 6506*
2504092271	Isoleucyl-tRNA synthetase (EC 6.1.1.5)	*Oscillatoria nigro-viridis PCC 7112*
2505171129	Isoleucyl-tRNA synthetase (EC 6.1.1.5)	*Microcoleus vaginatus PCC 9802*
2506346988	Isoleucyl-tRNA synthetase (EC 6.1.1.5)	*Microcoleus vaginatus FGP-2*
2508877648	Isoleucyl-tRNA synthetase (EC 6.1.1.5)	*Oscillatoria formosa PCC 6407*
2509423134	Isoleucyl-tRNA synthetase (EC 6.1.1.5)	*Oscillatoria acuminata PCC 6304*
2510101723	Isoleucyl-tRNA synthetase (EC 6.1.1.5)	*Geitlerinema sp. PCC 7105*
2510440960	Isoleucyl-tRNA synthetase (EC 6.1.1.5)	*Chamaesiphon minutus PCC 6605*
2609130917	Isoleucyl-tRNA synthetase (EC 6.1.1.5)	*Phormidium sp. OSCR*
640020071	leucyl-tRNA synthetase (EC 6.1.1.4)	*Lyngbya sp. CCY 8106*
2504685698	leucyl-tRNA synthetase (EC 6.1.1.4)	*Crinalium epipsammum PCC 9333*
2509509165	methionyl-tRNA synthetase (EC 6.1.1.10)	*Oscillatoria sp. PCC 10802*
2509746733	Valyl-tRNA synthetase	*Leptolyngbya sp. 2LT21S03 (Solid & 454 Clean assembly draft 1)*
637231684	valyl-tRNA synthetase (EC 6.1.1.9)	*Nostoc sp. PCC 7120*
641251708	valyl-tRNA synthetase (EC 6.1.1.9)	*Acaryochloris marina MBIC11017*
642603643	valyl-tRNA synthetase (EC 6.1.1.9)	*Nostoc punctiforme PCC 73102*
643584081	valyl-tRNA synthetase (EC 6.1.1.9)	*Cyanothece sp. PCC 7425*
646568479	valyl-tRNA synthetase (EC 6.1.1.9)	*Anabaena variabilis ATCC 29413*
647105916	valyl-tRNA synthetase (EC 6.1.1.9)	*Cylindrospermopsis raciborskii CS-505*
647108991	valyl-tRNA synthetase (EC 6.1.1.9)	*Raphidiopsis brookii D9*
647580186	valyl-tRNA synthetase (EC 6.1.1.9)	*Synechococcus sp. PCC 7335*
648051548	valyl-tRNA synthetase (EC 6.1.1.9)	*Nostoc azollae 0708*
2022830072	valyl-tRNA synthetase (EC 6.1.1.9)	*Leptolyngbya sp. JSC-1*
2501541529	valyl-tRNA synthetase (EC 6.1.1.9)	*Tolypothrix sp. PCC 7601*
2503245767	valyl-tRNA synthetase (EC 6.1.1.9)	*Planktothrix agardhii NIVA-CYA 126/8*
2503368123	valyl-tRNA synthetase (EC 6.1.1.9)	*Cyanobacterium stanieri PCC 7202*
2503637215	valyl-tRNA synthetase (EC 6.1.1.9)	*Halothece sp. PCC 7418*
2503741712	valyl-tRNA synthetase (EC 6.1.1.9)	*Nostoc sp. PCC 7107*
2503745801	valyl-tRNA synthetase (EC 6.1.1.9)	*Cyanobacterium aponinum PCC 10605*
2504093999	valyl-tRNA synthetase (EC 6.1.1.9)	*Calothrix sp. PCC 6303*
2504130158	valyl-tRNA synthetase (EC 6.1.1.9)	*Anabaena cylindrica PCC 7122*

2505768505	valyl-tRNA synthetase (EC 6.1.1.9)	*Fischerella sp. JSC-11*
2505799244	valyl-tRNA synthetase (EC 6.1.1.9)	*Calothrix sp. PCC 7507*
2506077272	valyl-tRNA synthetase (EC 6.1.1.9)	*Planktothrix NIVA-CYA406*
2506383335	valyl-tRNA synthetase (EC 6.1.1.9)	*Planktothrix sp. NIVA CYA 15*
2506395049	valyl-tRNA synthetase (EC 6.1.1.9)	*Planktothrix sp. 585*
2506414756	valyl-tRNA synthetase (EC 6.1.1.9)	*Planktothrix agardhii NIVA-CYA 34*
2506420479	valyl-tRNA synthetase (EC 6.1.1.9)	*Planktothrix prolifica NIVA-CYA 98*
2506480986	valyl-tRNA synthetase (EC 6.1.1.9)	*Lyngbya majuscula 3L*
2506492816	valyl-tRNA synthetase (EC 6.1.1.9)	*Anabaena sp. PCC 7108*
2506598839	valyl-tRNA synthetase (EC 6.1.1.9)	*Spirulina subsalsa PCC 9445*
2506610257	valyl-tRNA synthetase (EC 6.1.1.9)	*Spirulina major PCC 6313*
2507337115	valyl-tRNA synthetase (EC 6.1.1.9)	*Scytonema hofmanni UTEX 2349*
2507412169	valyl-tRNA synthetase (EC 6.1.1.9)	*Planktothrix sp. st147*
2507480205	valyl-tRNA synthetase (EC 6.1.1.9)	*Calothrix sp. PCC 7103*
2509431957	valyl-tRNA synthetase (EC 6.1.1.9)	*Microcoleus sp. PCC 7113*
2509553970	valyl-tRNA synthetase (EC 6.1.1.9)	*Dactylococcopsis salina PCC 8305*
2509768206	valyl-tRNA synthetase (EC 6.1.1.9)	*Cylindrospermum stagnale PCC 7417*
2509774928	valyl-tRNA synthetase (EC 6.1.1.9)	*Nodosilinea nodulosa PCC 7104*
2509782933	valyl-tRNA synthetase (EC 6.1.1.9)	*Microchaete sp. PCC 7126*
2509802406	valyl-tRNA synthetase (EC 6.1.1.9)	*Leptolyngbya boryana PCC 6306*
2509812648	valyl-tRNA synthetase (EC 6.1.1.9)	*Nostoc sp. PCC 7524*
2509841850	valyl-tRNA synthetase (EC 6.1.1.9)	*Leptolyngbya sp. PCC 7375*
2509875443	valyl-tRNA synthetase (EC 6.1.1.9)	*Geminocystis herdmanii PCC 6308*
2510027282	valyl-tRNA synthetase (EC 6.1.1.9)	*Calothrix desertica PCC 7102*
2510088921	valyl-tRNA synthetase (EC 6.1.1.9)	*Rivularia sp. PCC 7116*
2512635464	valyl-tRNA synthetase (EC 6.1.1.9)	*cyanobacterium PCC 7702*
2512980725	valyl-tRNA synthetase (EC 6.1.1.9)	*Fischerella sp. PCC 9431*
2512987564	valyl-tRNA synthetase (EC 6.1.1.9)	*Planktothrix prolifica NIVA-CYA 540 (Draft1)*
2514738238	valyl-tRNA synthetase (EC 6.1.1.9)	*Acaryochloris sp CCMEE 5410*
2516148082	valyl-tRNA synthetase (EC 6.1.1.9)	*Fischerella sp. PCC 9605*
2516860686	valyl-tRNA synthetase (EC 6.1.1.9)	*Anabaena circinalis AWQC131C*
2516864791	valyl-tRNA synthetase (EC 6.1.1.9)	*Anabaena circinalis AWQC310F*
2517064125	valyl-tRNA synthetase (EC 6.1.1.9)	*Fischerella sp. PCC 9339*
2517241608	valyl-tRNA synthetase (EC 6.1.1.9)	*Mastigocladopsis repens PCC 10914*
2517649263	valyl-tRNA synthetase (EC 6.1.1.9)	*Filamentous cyanobacterium ESFC-1*
2517690921	valyl-tRNA synthetase (EC 6.1.1.9)	*Leptolyngbya sp. PCC 6406*
2522376071	valyl-tRNA synthetase (EC 6.1.1.9)	*Cylindrospermopsis raciborskii CS-509*
2523878684	valyl-tRNA synthetase (EC 6.1.1.9)	*Planktothrix agardhii NIVA-CYA 56/3*
2529412579	valyl-tRNA synthetase (EC 6.1.1.9)	*Westiella intricata UH HT-29-1*
2529416758	valyl-tRNA synthetase (EC 6.1.1.9)	*Hapalosiphon welwitschii UH strain IC-52-3*
2550702003	valyl-tRNA synthetase (EC 6.1.1.9)	*Fischerella muscicola SAG 1427-1*
2550708624	valyl-tRNA synthetase (EC 6.1.1.9)	*Fischerella muscicola PCC 7414*
2550715971	valyl-tRNA synthetase (EC 6.1.1.9)	*Fischerella thermalis PCC 7521*
2550831074	valyl-tRNA synthetase (EC 6.1.1.9)	*Chlorogloeopsis fritschii PCC 9212*

2551962007	valyl-tRNA synthetase (EC 6.1.1.9)	*Scytonema hofmanni PCC 7110*
2551968109	valyl-tRNA synthetase (EC 6.1.1.9)	*Chlorogloeopsis fritschii PCC 6912*
2562230881	valyl-tRNA synthetase (EC 6.1.1.9)	*Anabaena sp. 90*
2563159503	valyl-tRNA synthetase (EC 6.1.1.9)	*Nodularia spumigena CCY9414*
2579004233	valyl-tRNA synthetase (EC 6.1.1.9)	*Leptolyngbya sp. Heron Island J*
2580947769	valyl-tRNA synthetase (EC 6.1.1.9)	*Richelia intracellularis HH01*
2586758929	valyl-tRNA synthetase (EC 6.1.1.9)	*Aphanizomenon flos-aquae NIES-81*
2588031065	valyl-tRNA synthetase (EC 6.1.1.9)	*Calothrix sp. 336/3*
2600482339	valyl-tRNA synthetase (EC 6.1.1.9)	*Planktothrix mougeotii NIVA-CYA 405*
2602458587	valyl-tRNA synthetase (EC 6.1.1.9)	*Planktothrix prolifica NIVA-CYA 406*
2602464450	valyl-tRNA synthetase (EC 6.1.1.9)	*Planktothrix agardhii NIVA-CYA 15*
2602532472	valyl-tRNA synthetase (EC 6.1.1.9)	*Planktothrix rubescens NIVA-CYA 407*
2609110326	valyl-tRNA synthetase (EC 6.1.1.9)	*Phormidesmis priestleyi ANA*
2619252232	valyl-tRNA synthetase (EC 6.1.1.9)	*Tolypothrix bouteillei Iicb1*
2619438059	valyl-tRNA synthetase (EC 6.1.1.9)	*Leptolyngbya sp. KIOST-1*
2622770592	valyl-tRNA synthetase (EC 6.1.1.9)	*Richelia intracellularis RC01*
