# Supplementary material for: Sub-Cellular Localization and Complex Formation by Aminoacyl-tRNA Synthetases in Cyanobacteria: Evidence for Interaction of Membrane-Anchored ValRS with ATP Synthase
Source: Front Microbiol. 2016 Jun 6;7:857. doi: 10.3389/fmicb.2016.00857 (PMC4893482; doi:10.3389/fmicb.2016.00857)
Supplement: Supplementary file 2 [file Table2.PDF]

Table S2. Cyanobacterial genomes containing two genes encoding $aaRSs^C$

Genome	$aaRS^C$-1	$aaRS^C$-2
Crinalium epipsammum PCC 9333	CysRS	LeuRS
Microcoleus vaginatus PCC 9802	GluRS	IleRS
Oscillatoria acuminata PCC 6304	GluRS	IleRS
Oscillatoria nigro-viridis PCC 7112	GluRS	IleRS
Trichodesmium erythraeum IMS101	GluRS	IleRS
Oscillatoria sp. PCC 10802	GluRS	MetRS
Spirulina major PCC 6313	ArgRS	ValRS
Spirulina subsalsa PCC 9445	ArgRS	ValRS
