# Supplementary material for: Sub-Cellular Localization and Complex Formation by Aminoacyl-tRNA Synthetases in Cyanobacteria: Evidence for Interaction of Membrane-Anchored ValRS with ATP Synthase
Source: Front Microbiol. 2016 Jun 6;7:857. doi: 10.3389/fmicb.2016.00857 (PMC4893482; doi:10.3389/fmicb.2016.00857)
Supplement: Supplementary file 3 [file Table3.PDF]

**Table S3.** Summary

<table>
<tr><td colspan="2">279 cyanobacterial genomes of the IMG Database were searched for the presence of aaRSs with CAAD as of 07/29/2015</td></tr>
<tr><td>2 genomes contain ArgRS with CAAD</td><td rowspan="7">102 genomes encode one or two aaRSs[c]</td></tr>
<tr><td>1 genome contains CysRS with CAAD</td></tr>
<tr><td>5 genomes contain GluRS with CAAD</td></tr>
<tr><td>11 genomes contain IleRS with CAAD</td></tr>
<tr><td>2 genomes contain LeuRS with CAAD</td></tr>
<tr><td>1 genome contains MetRS with CAAD</td></tr>
<tr><td>79 genones contain ValRS with CAAD</td></tr>
<tr><td colspan="2">36% of cyanobacterial species contain aaRSs with CAAD</td></tr>
</table>
