# Supplementary material for: Sub-Cellular Localization and Complex Formation by Aminoacyl-tRNA Synthetases in Cyanobacteria: Evidence for Interaction of Membrane-Anchored ValRS with ATP Synthase
Source: Front Microbiol. 2016 Jun 6;7:857. doi: 10.3389/fmicb.2016.00857 (PMC4893482; doi:10.3389/fmicb.2016.00857)
Supplement: Supplementary file 4 [file Table4.PDF]

Table S4: Proteins that co-purify with aaRSs.

	Protein name	MW	Annotation	Comments
**GFP-MetRS**	Alr2312	53097	Hypothetical protein<br>Putative zinc-dependent peptidase	
	Alr1845 (TrmB)	24848	tRNA (guanine-N(7)-)-methyltransferase	
	All1975	44600	Hypothetical protein	
	All0914	39188	Hypothetical protein, probable glycosyl transferase	
	All4337(TufA)	44680	Elongation Factor-Tu	
**GFP-AspRS**	Alr7308	32219	hypothetical protein<br>putative HNH nuclease	
	Alr3067	36956	hypothetical protein	
	Alr2954	20810	hypothetical protein<br>putative NUDIX hydrolase	
**GFP-αGlyRS**	Alr4111	79215	Glycyl-tRNA synthetase, beta subunit	In *Anabaena* GlyRS is a heterodimer composed of subunits α and β
**GFP-LysRS**	All4019	58291	Glucose-6-phosphate 1-dehydrogenase;	
**GFP-SerRS**	All4337(TufA)	44680	Elongation Factor-Tu	
	Alr3402(ndK)	16473	Nucleoside-diphosphate kinase	
**GFP-T1**	All4337(TufA)	44680	Elongation Factor-Tu	
**GFP-LeuRS**	Alr1041(GlpX)	36606	D-fructose 1,6-bisphosphatase class 2/sedoheptulose 1,7-bisphosphatase	
	Alr0529(CpcA)	17326	C-phycocyanin alpha chain	
	Alr0021(ApcA1)	17215	Allophycocyanin subunit alpha 1	
	All4337(TufA)	44680	Elongation Factor-Tu	
**GFP-GluRS**	Alr2691	78787	hypothetical protein	
	Alr5249	80693	hypothetical protein (putative nucleic acids binding protein)	
	Alr2784	92862	hypothetical protein (putative metal dependent phosphohydrolase)	
	Alr2791	132044	hypothetical protein (WD-repeat protein)	
	All7286	34743	hypothetical protein	
	Alr3115	114780	hypothetical protein (putative metallopeptidase)	
	Alr1146	94725	hypothetical protein (GTPase domain)	
	All0373	37983	putative serine/threonine protein phosphatase	
**GFP-αPheRS**	Alr4958(PheT)	89031	phenylalanyl-tRNA synthetase subunit beta;	PheRS is a $(\alpha,\beta)_2$ heterotetramer
**GFP-ArgRS**	Alr7552	102324	ATP-dependent RNA helicase	
	Alr7147	58826	Transposase	
	All3333	47813	Nitrate binding protein	
	All0862	34459	LysR family transcriptional regulator	
