# Supplementary material for: Sub-Cellular Localization and Complex Formation by Aminoacyl-tRNA Synthetases in Cyanobacteria: Evidence for Interaction of Membrane-Anchored ValRS with ATP Synthase
Source: Front Microbiol. 2016 Jun 6;7:857. doi: 10.3389/fmicb.2016.00857 (PMC4893482; doi:10.3389/fmicb.2016.00857)
Supplement: Supplementary file 5 [file Table5.PDF]

Table S5. Plasmids utilized in this work. Ap, ampicillin; Sm, spectinomycin; Sp, Streptomycin; Km, kanamycin

Plasmid	Resist.	Description	Ref.
pCJS48	Ap	Plasmid constructed by cloning in the pSparK-I vector (Invitrogen) a PCR fragment amplified with primers GFPMUT2-1F and GFPMUT2-2R encompassing the ORF of GFP-mut2	This work
pCJS49	Sm Sp	Plasmid constructed by cloning a PCR fragment that contains the *Anabaena* P*petE* promoter (generated with primers PPETE-3F and PPETE-2R) between sites EcoRI and StuI of pCJS48.	This work
pCJS51	Sm Sp	Plasmid constructed by cloning a PCR fragment containing the P*petE-gfpmut2* fusion (generated with primers PCJS49-1F and PCJS49-1R) in the EcoRI site of vector pCSV3	This work
pCJS52	Sm Sp	Plasmid derived of pCJS51 containing between the BamHI and XhoI sites a PCR fragment (generated with primers A7120-ASPRS-1F and A7120-ASPRS-1R) encompassing the ORF encoding AspRS in *Anabaena*. Suitable for the controlled expression of the GFP:-AspRS fusion protein in *Anabaena*	This work
pCJS53	Sm Sp	Plasmid derived of pCJS51 containing between the BamHI and XhoI sites a PCR fragment (generated with primers A7120-METRS-1F and A7120-METRS-1R) encompassing the ORF encoding MetRS in *Anabaena*. Suitable for the controlled expression of the GFP-MetRS fusion protein in *Anabaena*	This work
pCJS55	Sm Sp	Plasmid derived of pCJS51 containing between the BamHI and XhoI sites a PCR fragment (generated with primers A7120-GLURS-2F and A7120-GLURS-3R) encompassing the ORF encoding GluRS in *Anabaena*. Suitable for the controlled expression of the GFP-GluRS fusion protein in *Anabaena*	This work
pCJS56	Sm Sp	Plasmid derived of pCJS51 containing between the BamHI and XhoI sites a PCR fragment (generated with primers A7120-ARGRS-1F and A7120-ARGRS-1R) encompassing the ORF encoding ArgRS in *Anabaena*. Suitable for the controlled expression of the GFP-ArgRS fusion protein in *Anabaena*	This work
pCJS57	Sm Sp	Plasmid derived of pCJS51 containing between the BamHI and XhoI sites a PCR fragment (generated with primers A7120-PHERSALPHA-1F and A7120-PHERSALPHA-1R) encompassing the ORF encoding the alpha subunit of PheRS in *Anabaena*. Suitable for the controlled expression of the GFP-PheRS(α) fusion protein in *Anabaena*	This work
pCJS58	Sm Sp	Plasmid derived of pCJS51 containing between the BamHI and XhoI sites a PCR fragment (generated with primers A7120-CYSRS-1F and A7120-CYSRS-1R) encompassing the ORF encoding the CysRS in *Anabaena*. Suitable for the controlled expression of the GFP-CysRS fusion protein in *Anabaena*	This work
pCJS59	Sm Sp	Plasmid derived of pCJS51 containing between the BamHI and XhoI sites a PCR fragment (generated with primers A7120-ILERS-1F and A7120-ILERS-1R) encompassing the ORF encoding the IleRS in *Anabaena*. Suitable for the controlled expression of the GFP-IleRS fusion protein in *Anabaena*	This work
pCJS60	Sm Sp	Plasmid derived of pCJS51 containing between the BamHI and XhoI sites a PCR fragment (generated with primers A7120-LEURS-1F and A7120-LEURS-1R) encompassing the ORF encoding the LeuRS in *Anabaena*. Suitable for the controlled expression of the GFP-LeuRS fusion protein in *Anabaena*	This work
pCJS61	Sm Sp	Plasmid derived of pCJS51 containing between the BamHI and XhoI sites a PCR fragment (generated with primers A7120-TRPRS-1F and A7120-TRPRS-1R) encompassing the ORF encoding the TrpRS in *Anabaena*. Suitable for the controlled expression of the GFP-TrpRS fusion protein in *Anabaena*	This work
pCJS62	Sm Sp	Plasmid derived of pCJS51 containing between the BamHI and XhoI sites a PCR fragment (generated with primers A7120-TYRRS-1F and A7120-TYRRS-1R) encompassing the ORF encoding the TyrRS in *Anabaena*. Suitable for the controlled expression of the GFP-TyrRS fusion protein in *Anabaena*	This work
pCJS63	Sm Sp	Plasmid derived of pCJS51 containing between the BamHI and XhoI sites a PCR fragment (generated with primers A7120-HISRS-1F and A7120-HISRS-1R) encompassing the ORF encoding the HisRS in *Anabaena*. Suitable for the controlled expression of the GFP-HisRS fusion protein in *Anabaena*	This work
pCJS64	Sm Sp	Plasmid derived of pCJS51 containing between the BamHI and XhoI sites a PCR fragment (generated with primers A7120-THRRS1-1F and A7120-THRRS1-1R) encompassing the ORF encoding T1 in *Anabaena*. Suitable for the controlled expression of the GFP-T1 fusion protein in *Anabaena*	This work
pCJS65	Sm Sp	Plasmid derived of pCJS51 containing between the BamHI and XhoI sites a PCR fragment (generated with primers A7120-PRORS-1F and A7120-PRORS-1R) encompassing the ORF encoding ProRS in *Anabaena*. Suitable for the controlled expression of the GFP-ProRS fusion protein in *Anabaena*	This work
pCJS66	Sm Sp	Plasmid derived of pCJS51 containing between the BamHI and XhoI sites a PCR fragment (generated with primers A7120-SERRS-1F and A7120-SERRS-1R) encompassing the ORF encoding SerRS in *Anabaena*. Suitable for the controlled expression of the GFP-SerRS fusion protein in *Anabaena*	This work
pCJS67	Sm Sp	Plasmid containing a PCR fragment encompassing the *Anabaena* sp. PCC 7120 *argRSC* ORF amplified with primers A7120-argRSC-7F y A7120-argRSC-7R and cloned in the BamHI site of pCJS51.	This work
pCJS68	Sm Sp	Plasmid derived of pCJS51 containing between the BamHI and XhoI sites a PCR fragment (generated with primers A7120-THRRS2-4F and A7120-THRRS2-6R) encompassing the ORF encoding T2 in *Anabaena*. Suitable for the controlled expression of the GFP-T2 fusion protein in *Anabaena*	This work
pCJS69	Sm Sp	Plasmid derived of pCJS51 containing between the BamHI and XhoI sites a PCR fragment (generated with primers A7120-ASNRS-1F and A7120-ASNRS-1R) encompassing the ORF encoding AsnRS in *Anabaena*. Suitable for the controlled expression of the GFP-AsnRS fusion protein in *Anabaena*	This work
	Sm	Plasmid derived of pCJS51 containing between the BamHI and XhoI sites a PCR fragment	

pCJS70	Sp	(generated with primers A7120-LYSRS-1F and A7120-LYSRS-1R) encompassing the ORF encoding LysRS in *Anabaena*. Suitable for the controlled expression of the GFP-LysRS fusion protein in *Anabaena*	This work
pCJS71	Sm<br>Sp	Plasmid derived of pCJS51 containing between the BamHI and XhoI sites a PCR fragment (generated with primers A7120-ALARS-1F and A7120-ALARS-1R) encompassing the ORF encoding AlaRS in *Anabaena*. Suitable for the controlled expression of the GFP-AlaRS fusion protein in *Anabaena*	This work
pCJS72	Sm<br>Sp	Plasmid derived of pCJS51 containing between the BamHI and XhoI sites a PCR fragment (generated with primers A7120-GLYRSALPHA-1F and A7120-GLYRSALPHA-1R) encompassing the ORF encoding the alpha subunit of GlyRS in *Anabaena*. Suitable for the controlled expression of the GFP-GlyRS(α) fusion protein in *Anabaena*	This work
pCJS73	Sm<br>Sp	Plásmido que contiene un fragmento de PCR que comprende 750 pb del gen *argRSC* de *Anabaen*a sp. PCC 7120, amplificado con los cebadores A7120-argRSC-8F y A7120-argRSC-8R, clonado en el sitio XhoI de pCJS51.	This work
pCJS74	Sm<br>Sp	Plasmid derived of pCJS51 containing between the BamHI and XhoI sites a PCR fragment (generated with primers A7120-VALRS-1F and A7120-VALRS-1R) encompassing the ORF encoding ValRS in *Anabaena*. Suitable for the controlled expression of the GFP-ValRS fusion protein in *Anabaena*	This work
pCJS83	Sm<br>Sp	Plasmid derived of pCJS51 containing between the BamHI and XhoI sites a PCR fragment (generated with primers LY8106-LEURS-1F and LY8106-LEURS-1R) encompassing the ORF encoding LeuRS in *Lyngbya* sp. PCC 8106. Suitable for the controlled expression of the GFP-(Lyngbya)LeuRS fusion protein in *Anabaena*	This work
pCE50	Km	Plasmid for the controlled expression of ValRS-6His in *Escherichia coli* C41(DE3)	Olmedo-Verd et al, 2011
pCE53	Km	Plasmid for the controlled expression of ValRSΔ^{C} -6His in *Escherichia coli* C41(DE3)	Olmedo-Verd et al, 2011
pCE56	Sm<br>Sp	Plasmid for the controlled expression of ValRS-6His in *Anabaena*	Olmedo-Verd et al, 2011
pCE57	Sm<br>Sp	Plasmid for the controlled expression of ValRS-Gfp in *Anabaena*	Olmedo-Verd et al, 2011
pCE58	Sm<br>Sp	Plasmid for the controlled expression of ValRSΔ^{C}-6His GFP in *Anabaena*	Olmedo-Verd et al, 2011
pCE59	Sm<br>Sp	Plasmid for the controlled expression of ValRSΔ^{C}-GFP in *Anabaena*	Olmedo-Verd et al, 2011
