# Supplementary material for: Sub-Cellular Localization and Complex Formation by Aminoacyl-tRNA Synthetases in Cyanobacteria: Evidence for Interaction of Membrane-Anchored ValRS with ATP Synthase
Source: Front Microbiol. 2016 Jun 6;7:857. doi: 10.3389/fmicb.2016.00857 (PMC4893482; doi:10.3389/fmicb.2016.00857)
Supplement: Supplementary file 6 [file Table6.PDF]

Table S6. Oligonucleotides utilized in this work

Name	Sequence (5'-3')
GFP-mut2-1F	GAAGGAGCATATGAGTAAAGGAGAAGAAC
GFPmut2-2R	CCTCCTCCTTTGTATAGTTCATCCATGCC
PpetE-3F	CACAGAATTCAGAACACAGTACTC
PpetE-2R	TTCAAGGCCTTCTCCTAACCTG
pCJS49-1F	AACGAATTCAGAACACAGTACTC
pCJS49-1R	ATAGAATTCTCAAGCTATGCATC
A7120-AspRS-1F	TTAGGATCCATGCGAACTCAC
A7120-AspRS-1R	CATTCTCGAGAATCCAGGTATC
A7120-MetRS-1F	TTAGGATCCATGAATCTAGTG
A7120-MetRS-1R	GAACTCGAGCTGAATTAGCTT
A7120-GluRS-2F	ACTGGATCCGTTAGAGTCAGAATC
A7120-GluRS-3R	GGCTCGAGATTGCCCACCAC
A7120-ArgRS-1F	TATGGATCCGCTACACAAGAAC
A7120-ArgRS-1R	CTTCTCGAGTTACTTTATCAATAGC
A7120-PheRSalpha-1F	CTAAGGATCCATGACTAGCAAC
A7120-PheRSalpha-1R	GAATGCTCGAGTCTGAGTTTTTC
A7120-CysRS-1F	GTTGGATCCCTAACGATTTAC
A7120-CysRS-1R	GTTCTCGAGGTAATAGCTTTG
A7120-IleRS-1F	GCTGGATCCGAAACTGGAAGTTAC
A7120-IleRS-1R	GATGTCTCGAGCTACAGGTGATTG
A7120-LeuRS-1F	GAAGGATCCGTGGACTCCCGATAC
A7120-LeuRS-1R	GACTCGAGGAAAGACAGCTTTGG
A7120-TrpRS-1F	CTAGGATCCATGGGTAAGCAG
A7120-TrpRS-1R	TTGCTCGAGGCATAAATTAAAC
A7120-TyrRS-1F	GTGGATCCGAAAATTTTTCTTGG
A7120-TyrRS-1R	CCCCTCGAGCCTCTAAGGTAC
A7120-HisRS-1F	GAGGATCCGCAAAAAACGAC
A7120-HisRS-1R	TACTCGAGGCACAGAAAAGAG
A7120-ThrRS1-1F	CTAGGATCCCAGCAGCTAATG
A7120-ThrRS1-1R	TTCTCGAGGGGTCATACCCAG
A7120-ProRS-1F	GAAGGATCCATGCGACTGTCAC
A7120-ProRS-1R	TTTCTCGAGCTGCATTCCTCG
A7120-SerRS-1F	GAAGGATCCGTGCTGGATATTAAG
A7120-SerRS-1R	AACTCGAGAGTGCTTTGTATCAG
A7120-argRSC-7F	CTTGGATCCTAACAGCGTCAG
A7120-argRSC-7R	CAGTGGATCCTGAGTGACTTCTG
A7120-ThrRS2-4F	ATTGGATCCGTCAGTTCCTTAAC
A7120-ThrRS2-6R	CTGCTCGAGGATTTTGTATTTG
A7120-AsnRS-1F	GGGGATCCATGGTAAATCGAC
A7120-AsnRS-1R	CACCTCGAGTTATTTGACGACTG
A7120-LysRS-1F	GTTCGGATCCATGTCGGAAGAAG
A7120-LysRS-1R	GACCTCGAGAACGTCAAACCC
A7120-AlaRS-1F	CTGGATCCTCTTCAAACCCG
A7120-AlaRS-1R	CCTCTCGAGTTAATTTCTCACGC
A7120-GlyRSalpha-1F	GAGGATCCAGTATGAATTTTC
A7120-GlyRSalpha-1R	GGCTCGAGAAATATTACTAAG
A7120-ValRS-1F	C AGAGGATCCGCAACTATAACC
A7120-ValRS-1R	CTAAATCTCGAGGGTGATGCCTAC
A7120-argRSC-8F	GCCCCTCGAGAAAACCAAGTGTATC
A7120-argRSC-8R	CAAAGTCGACACTGGCTCCAAAAAG
LY8106-LeuRS-1F	GTTGGATCCGTGGAGTCTCG
LY8106-LeuRS-1R	GACTCGAGACGGGATTATTTAG
