# Supplementary material for: Sub-Cellular Localization and Complex Formation by Aminoacyl-tRNA Synthetases in Cyanobacteria: Evidence for Interaction of Membrane-Anchored ValRS with ATP Synthase
Source: Front Microbiol. 2016 Jun 6;7:857. doi: 10.3389/fmicb.2016.00857 (PMC4893482; doi:10.3389/fmicb.2016.00857)
Supplement: Supplementary file 7 [file Presentation1.PDF]

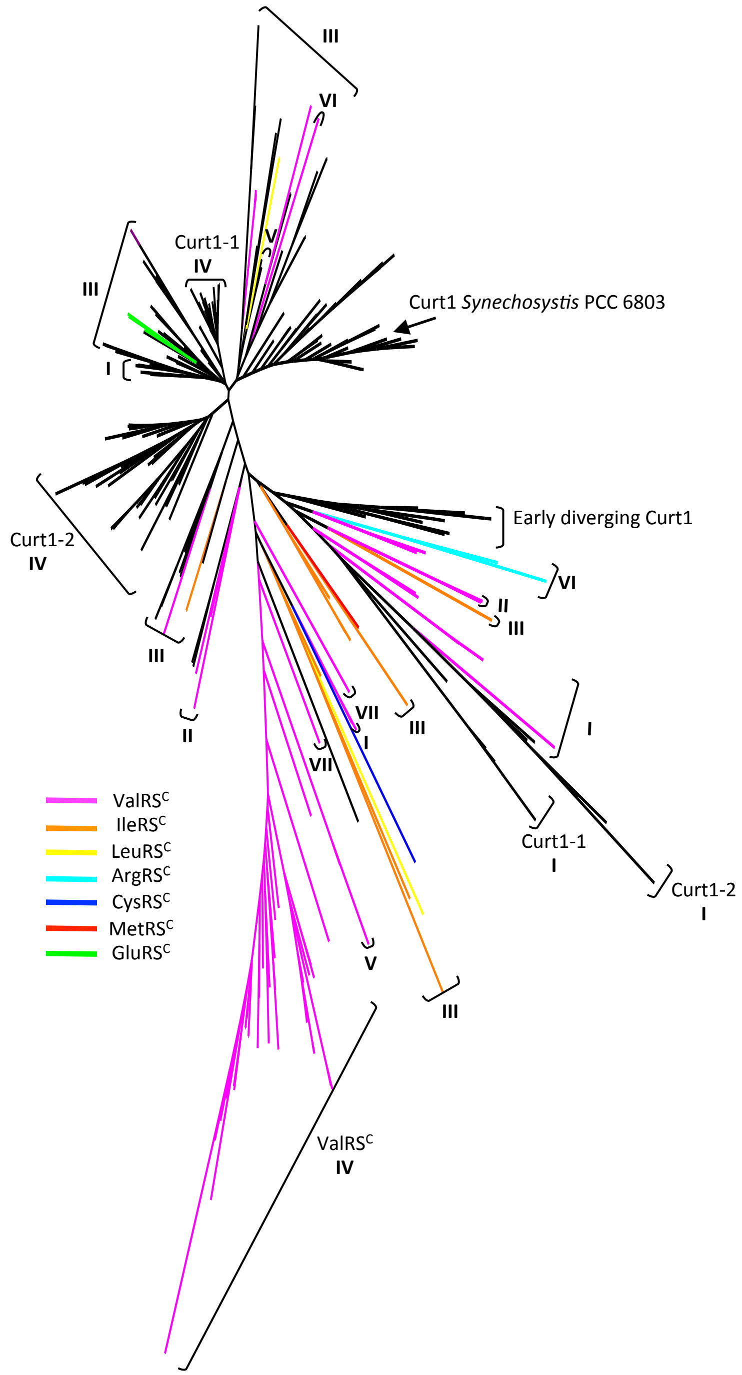

Figure S1. Phylogeny of Curt1/CAAD from cyanobacteria.
Branch colors identify the type of aaRS^{C} to which each CAAD belongs (as in Fig 1B), whereas brackets mark the cluster of organisms, as assigned in the guide tree of cyanobacteria (Fig. 1A). In the case of cluster IV (grouping all heterocystous cyanobacteria), the two Curt1 proteins (Curt1-1 and Curt1-2) encoded in the genome and CAAD are identified to evidence branch length differences and monophyly of each paralog (Curt1-1 and Curt1-2 at the top and ValRS^{C} at the botton). For cluster I (marine *Prochlorococcus* and *Synechococcus* clade), the two Curt1 proteins (Curt1-1and Curt1-2) encoded in the genome are identified to note that, in contrast to cluster IV paralogs, they appear during the expansion of the clade. Phylogeny was inferred from 307 homologous sequences retrieved from Refseq-NCBI database (as in March 2016) using CAAD of ValRS as query and PSI-BLAST searching tool until convergence (4 iterations). Sequences were aligned and trimmed using sequentially MAFFT 7 (G-INS-i leaving gappy regions)(Katoh & Standley, 2014) and BMGE 1.1 (Criscuolo & Gribaldo, 2010). Phylogeny was reconstructed using Fasttree 2.1.7 (WAG-CAT model)(Price, Dehal, & Arkin, 2010).

Criscuolo, A., & Gribaldo, S. (2010). BMGE (Block Mapping and Gathering with Entropy): a new software for selection of phylogenetic informative regions from multiple sequence alignments. *BMC Evol Biol, 10*, 210. doi: 10.1186/1471-2148-10-210

Katoh, K., & Standley, D. M. (2014). MAFFT: iterative refinement and additional methods. *Methods Mol Biol, 1079*, 131-146. doi: 10.1007/978-1-62703-646-7_8

Price, M. N., Dehal, P. S., & Arkin, A. P. (2010). FastTree 2--approximately maximum-likelihood trees for large alignments. *PLoS One, 5*(3), e9490. doi: 10.1371/journal.pone.0009490
