# Supplementary material for: Sub-Cellular Localization and Complex Formation by Aminoacyl-tRNA Synthetases in Cyanobacteria: Evidence for Interaction of Membrane-Anchored ValRS with ATP Synthase
Source: Front Microbiol. 2016 Jun 6;7:857. doi: 10.3389/fmicb.2016.00857 (PMC4893482; doi:10.3389/fmicb.2016.00857)
Supplement: Supplementary file 8 [file Presentation2.PDF]

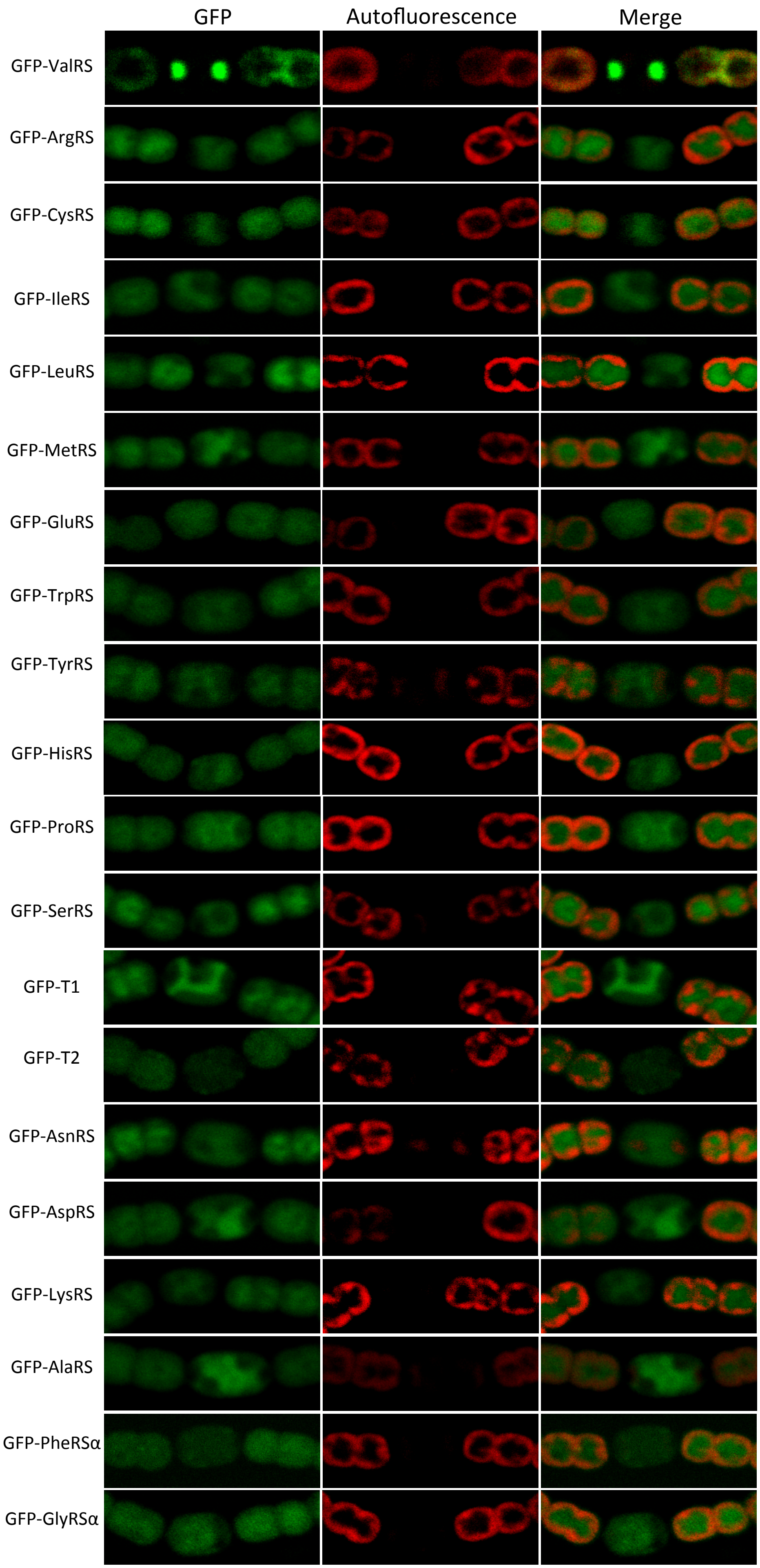

Fig S2. Localization of GFP-aaRS fusions in heterocysts of *Anabaena*.
Cells expressing the fusion protein indicated at the left of each panel were cultured in $BG11_0$ medium (lacking a source of combined nitrogen) for 24 hours and analyzed by confocal fluorescence microscopy. In each panel the central cell with low auto-fluorescence corresponds to a heterocyst and the flanking cells are vegetative cells.
