# Supplementary material for: Sub-Cellular Localization and Complex Formation by Aminoacyl-tRNA Synthetases in Cyanobacteria: Evidence for Interaction of Membrane-Anchored ValRS with ATP Synthase
Source: Front Microbiol. 2016 Jun 6;7:857. doi: 10.3389/fmicb.2016.00857 (PMC4893482; doi:10.3389/fmicb.2016.00857)
Supplement: Supplementary file 9 [file Presentation3.PDF]

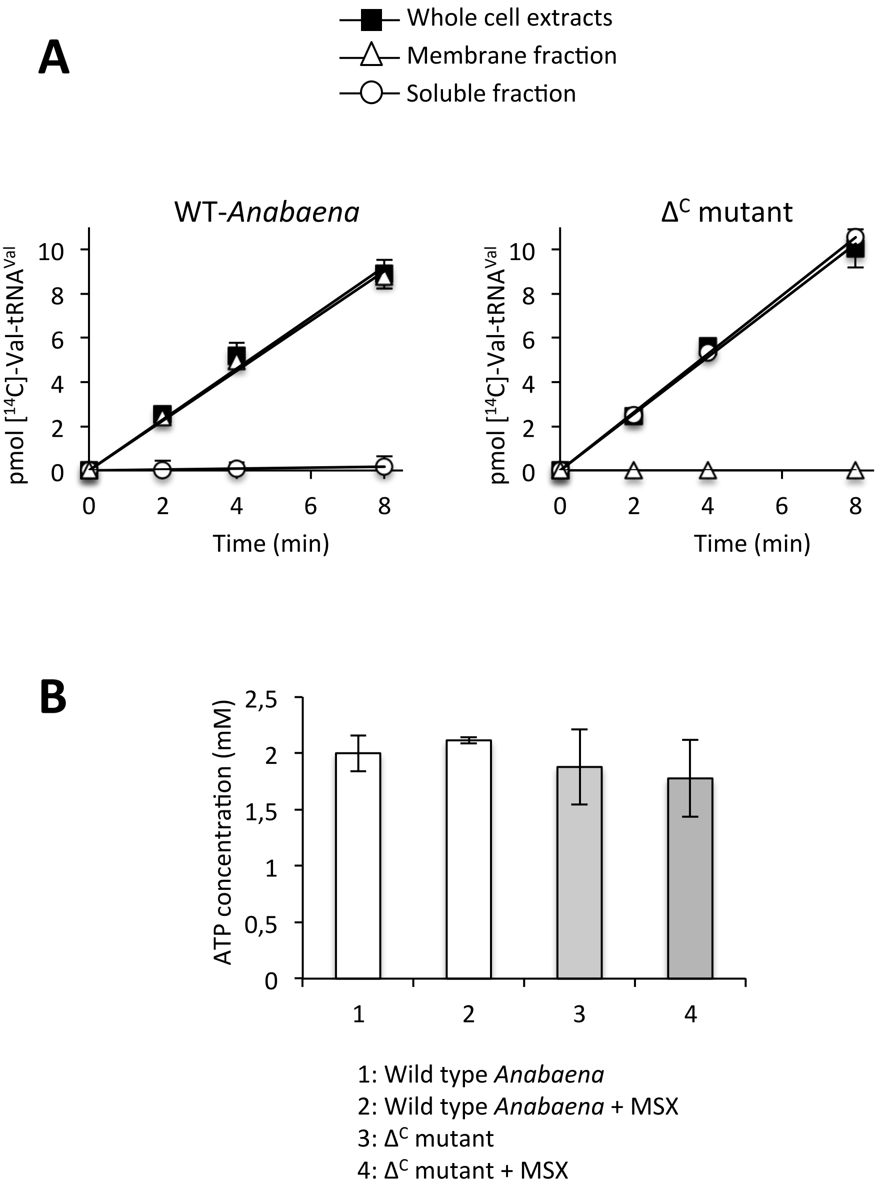


**Fig. S3.** Impact of the ValRS$^{C}$-ATP synthase interaction.
(A) Aminoacylation assays with cell fractions from wild-type cells and $\Delta^{C}$ cells. Values are the mean aminoacylation activity of three independent cultures and error bars correspond to the standard deviation. (B) ATP content of wild-type cells and $\Delta^{C}$ cells growing under standard conditions or after incubation with MSX for 4h as indicated. Values are the mean ATP concentration of three independent cultures and error bars correspond to the standard deviation.
